# Neurotensin-neurotensin receptor 2 signaling in adipocytes suppresses food intake through regulating ceramide metabolism

**DOI:** 10.1038/s41422-024-01038-8

**Published:** 2025-01-03

**Authors:** Wei Fu, Yuanting Lai, Kexin Li, Yue Yang, Xiao Guo, Qifan Gong, Xiaofeng Zhou, Liying Zhou, Cenxi Liu, Zhi Zhang, Jisun So, Yufeng Zhang, Lin Huang, Guangxing Lu, Chuanyou Yi, Qichu Wang, Chenyu Fan, Chao Liu, Jiaxing Wang, Haiyi Yu, Yimin Zhao, Tao Huang, Hyun Cheol Roh, Tiemin Liu, Huiru Tang, Jianping Qi, Ming Xu, Yan Zheng, He Huang, Jin Li

**Affiliations:** 1 State Key Laboratory of Genetic Engineering, School of Life Sciences, Institute of Metabolism and Integrative Biology, Human Phenome Institute and Zhongshan Hospital, Fudan University, Shanghai, China; 2 Department of Endocrinology, The First Affiliated Hospital and Clinical Medicine College, Henan University of Science and Technology, Luoyang, Henan China; 3 National Center for Clinical Research of Metabolic Diseases, Luoyang Center for Endocrinology and Metabolism, Luoyang, Henan China; 4 Diabetic Nephropathy Academician Workstation of Henan Province, Luoyang, Henan China; 5 Department of Biochemistry and Molecular Biology, Indiana University School of Medicine, Indianapolis, IN USA; 6 Department of Cardiology and Institute of Vascular Medicine, Peking University Third Hospital; State Key Laboratory of Vascular Homeostasis and Remodeling, Peking University; NHC Key Laboratory of Cardiovascular Molecular Biology and Regulatory Peptides, Peking University, Beijing, China; 7 Department of Epidemiology and Biostatistics, School of Public Health, Peking University, Beijing, China

**Keywords:** Extracellular signalling molecules, Metabolomics

## Abstract

Neurotensin (NTS) is a secretory peptide produced by lymphatic endothelial cells. Our previous study revealed that NTS suppressed the activity of brown adipose tissue via interactions with NTSR2. In the current study, we found that the depletion of *Ntsr2* in white adipocytes upregulated food intake, while the local treatment of NTS suppressed food intake. Our mechanistic study revealed that suppression of NTS-NTSR2 signaling enhanced the phosphorylation of ceramide synthetase 2, increased the abundance of its products ceramides C20–C24, and downregulated the production of GDF15 in white adipose tissues, which was responsible for the elevation of food intake. We discovered a potential causal and positive correlation between serum C20–C24 ceramide levels and human food intake in four populations with different ages and ethnic backgrounds. Together, our study shows that NTS-NTSR2 signaling in white adipocytes can regulate food intake via its direct control of lipid metabolism and production of GDF15. The ceramides C20–C24 are key factors regulating food intake in mammals.

## Introduction

Adipose tissue is one of the largest organs and a major regulator of metabolic homeostasis in mammals. Though its primary function is lipid storage, it affects metabolism via various routes. Several studies have demonstrated that adipose tissue is an important endocrine organ. Adipose tissue-derived secretary proteins, from either the adipocytes or stromal vascular fraction (SVF), tightly modulate the metabolic activity in many organs. One such well-known factor is leptin, which is an adipose tissue-derived secretory protein that regulates multiple aspects of metabolism via interactions with the leptin receptor.^1,2^ Adipose tissue also can affect the physiological function of skeletal muscle via the secretory protein myostatin.^3^ In addition to endocrine activity, adipose tissue-derived secretory proteins can regulate metabolic homeostasis via autocrine or paracrine signaling as well.^4^

Neurotensin (NTS) is a 13-amino acid secretory peptide with diverse functions that acts upon both the central nervous system (CNS) and peripheral tissues. Due to its very short half-life in the circulation,^5,6^ the native NTS can only exert its functions via the paracrine or autocrine mode. NTS was previously known to be produced by neurons and intestinal N cells.^7–9^ Two G protein-coupled receptors (GPCRs), NTSR1 and NTSR2, have been discovered to specifically interact with NTS.^10,11^ The type I membrane glycoprotein Sortilin (SORT1 or NTSR3) also interacts with NTS, though its downstream signaling is largely uncharacterized.^12–14^ Many of these studies attributed the decreases in food intake to the local and direct interaction between NTS and cells in the CNS, as intracerebroventricular (ICV) injection of NTS was sufficient to reduce food intake.^15–18^ The feeding behavior changes induced by NTS treatment is likely mediated by NTSR1, though the role of NTSR2 cannot be excluded.^19–22^

As far as we know, it is unknown whether NTS can regulate food intake through direct interactions with peripheral tissues or organs in physiological conditions. One possible reason is that for a long period, people believe that NTS is only produced by either the CNS or intestinal N cells. Though the results of ICV injection indicated that NTS could regulate food intake via direct interactions with the CNS, it did not rule out the possibility that NTS could also regulate food intake via interacting with peripheral tissues in physiological conditions.

With single-cell RNA-sequencing (scRNA-seq) analysis, we have determined that NTS was also produced specifically by lymphatic endothelial cells (LECs) as an anti-thermogenic peptide in brown adipose tissues (BATs)^23^ and a pro-lipid absorption peptide regulating the development of atherosclerosis in intestines.^24–26^ *Ntsr2*, but not *Ntsr1*, is widely expressed in brown adipocytes, beige adipocytes and white adipocytes.^27^ Transient knockdown experiments have indicated that NTS exerts its anti-thermogenic effects via the interactions with NTSR2 in brown adipose tissues through paracrine signaling. However, the cell-type-specific effects and long-term physiological functions of NTSR2, especially how it functions in the white adipocytes, are still unknown.

Ceramide is a kind of lipid with diverse biological functions. The metabolism of ceramide is extremely complicated, as these lipids can be synthesized and degraded by a large collection of enzymes in various pathways.^28^ It has been reported that depletion of ceramide synthetase 2 (*CerS2*) led to the downregulation of ceramides C20–C24 and upregulation of C16 due to compensatory effects in the liver.^29–33^ In addition, it has been demonstrated that ceramides C20–C24 are negative regulators of the unfolded protein response (UPR).^34,35^ The UPR, as a part of the integrative stress response, may affect the production of growth differentiation factor 15 (GDF15), a secretory protein.^36,37^ On one hand, GDF15 treatment suppresses food intake and decreases body weight via interacting with its receptor, GFRAL, in the area postrema/nucleus of the solitary tract region of the CNS; on the other hand, depletion of *Gdf15* or *Gfral* gene leads to increases in food intake and body weight.^38–47^ However, the mechanism to regulate the production and physiological functions of ceramides C20–C24 has not been elucidated.

In the current study, we established a brown/beige adipocyte-specific knockout (KO) mouse model for *Ntsr2* (*Ucp1-cre*::*Ntsr2*^*flox/flox*^, referred to as *Ntsr2* BKO mice), which exhibited an elevated energy expenditure and lower body weight when fed by a high-fat diet (HFD). By contrast, the adipocyte-specific *Ntsr2* depletion (*Adipoq-cre*::*Ntsr2*^*flox/flox*^, referred to *Ntsr2* AKO mice) presented the elevation of food intake, but the local treatment of NTS induced remarkable decreases of food intake in an NTSR2-dependent manner. The NTS-NTSR2 signaling controlled the synthesis of ceramides C20–C24, but not C16, by suppressing the phosphorylation of CerS2 via RhoA but not canonical CK2 kinase in white adipocytes. The suppression of ceramides C20–C24 production in adipose tissue by depleting *CerS2* downregulated food intake, but the local treatment of ceramides C20–C24 in vivo upregulated food intake. The ceramide-UPR-dependent production of GDF15 mediated a cross-talk between the NTS-NTSR2 signaling in the adipose tissue and food intake. We confirmed that the serum abundance of ceramides C20–C24 was positively correlated to energy intake in both children and adults. With Mendelian randomization analysis, we discovered a potential causal relation between ceramides C20–C24 levels and food intake in humans. The current study provides the first evidence for NTS-NTSR2 signaling to control food intake by directly regulating the ceramide metabolism in adipose tissues.

## Results

### Brown/beige adipocyte-specific depletion of *Ntsr2* increases energy expenditure

We first generated *Ntsr2*^*flox/flox*^ mice by knocking in the *LoxP* cassette before the first exon and after the last exon of the *Ntsr2* gene. By crossing the *Ntsr2*^*flox/flox*^ mouse with a *Ucp1-cre* mouse, the *Ntsr2* BKO mouse model (*Ucp1-cre*::*Ntsr2*^*flox/flox*^) was established and phenotypically characterized (Supplementary information, Fig. S1a). The *Ntsr2* depletion efficiency and specificity in BATs were validated by RT-qPCR (Supplementary information, Fig. S1b). The body weight and food intake of *Ntsr2* BKO mice and control mice (*Ntsr2*^*flox/flox*^ mice) fed by a chow diet were comparable (Supplementary information, Fig. S1c, d). Notably, *Ntsr2* BKO mice had a lower body weight than control mice when they were fed by HFD (Supplementary information, Fig. S1e). More lean mass and less fat mass were observed in *Ntsr2* BKO mice (Supplementary information, Fig. S1f). *Ntsr2* BKO mice also had a better performance in the glucose tolerance test (GTT) (Supplementary information, Fig. S1g) and insulin tolerance test (ITT) (Supplementary information, Fig. S1h).

The Comprehensive Lab Animal Monitoring System (CLAMS) analysis revealed that *Ntsr2* BKO mice presented a higher O_2_ consumption rate (Supplementary information, Fig. S1i) and CO_2_ production rate (Supplementary information, Fig. S1j), indicating an enhancement of energy expenditure. *Ntsr2* BKO mice also had increased expression of thermogenic genes in BATs but not in inguinal white adipose tissues (iWATs) (Supplementary information, Fig. S1k, l). By contrast, the physical activity and fecal energy between *Ntsr2* BKO mice and control mice were comparable (Supplementary information, Fig. S1m, n). It is widely observed that elevated thermogenesis only has effects on the body weight of obese but not young and lean mice.^48,49^ These results indicated that NTSR2 in the BATs had significant impacts on thermogenesis and development of diet-induced obesity (DIO).

### NTS-NTSR2 signaling in the white adipocytes regulates food intake

**Fig. 1:**
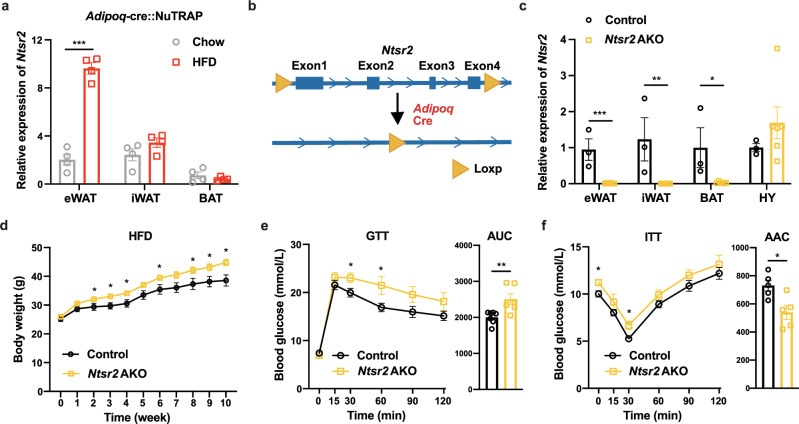
Depletion of *Ntsr2* in the adipocytes induced body weight elevation. **a** The expression of *Ntsr2* in the adipocyte-specific transcriptome of lean and obese mice (*n* = 4). **b** Scheme of the establishment of adipocyte-specific *Ntsr2* KO mouse strain. **c** KO efficiency and specificity of the *Ntsr2* gene (*n* = 3–6). **d**–**f** The body weight (**d**), GTT (**e**) and ITT (**f**) of mice fed by HFD (*n* = 5–7). *P < 0.05; **P < 0.01; ***P < 0.001.

By employing the transcriptomic analysis of adipocyte-specific mRNA isolated by translating ribosome affinity purification (TRAP),^27,50,51^ we found that the *Ntsr2* expression was dramatically upregulated by HFD, specifically in epididymal WATs (eWATs) (Fig. 1a). We therefore further explored the functions of NTSR2 in white adipocytes. By crossing *Ntsr2*^*flox/flox*^ mice with an *Adipoq-cre* mouse, the *Ntsr2* AKO mouse model was established and phenotypically characterized (Fig. 1b). *Ntsr2* depletion efficiency and specificity in the adipocytes and adipose tissues were validated by RT-qPCR (Fig. 1c; Supplementary information, Fig. S2a, b). The body weight of *Ntsr2* AKO mice and control mice (*Ntsr2*^*flox/flox*^ mice) fed by a chow diet was comparable (Supplementary information, Fig. S2c). But *Ntsr2* AKO mice had a higher body weight than control mice when fed by HFD (Fig. 1d). Interestingly, a similar elevation of body weight was observed in *Ntsr2* AKO mice when they were kept in the thermoneutral condition (a 12-h light/12-h dark cycle at constant 30 °C with free access to food and water) (Supplementary information, Fig. S2d). There is a trend of increased fat mass and lean mass in *Ntsr2* AKO mice (Supplementary information, Fig. S2e). *Ntsr2* AKO mice also had a worse performance in the GTT (Fig. 1e) and ITT (Fig. 1f).

We then explored the reasons for the body weight changes in *Ntsr2* AKO mice. It is known that impaired lipolysis might be an important biological process contributing to the development of DIO. However, we found that *Ntsr2* AKO rather led to the elevation of lipolysis-related proteins p-HSL and ATGL in the eWAT (Supplementary information, Fig. S2f). The concentration of non-esterified fatty acids (NEFAs) in serum was also elevated in *Ntsr2* AKO mice upon fasting (Supplementary information, Fig. S2g). To investigate whether the changes in lipolysis were due to the cell-autonomous effects of the NTS-NTSR2 signaling, we treated the primary adipocytes derived from SVF with recombinant NTS peptides. NTS treatment decreased the abundance of lipolysis-related proteins ATGL and p-HSL (Supplementary information, Fig. S2h). It also prevented lipolysis, determined by the reduced release of NEFA from adipocytes (Supplementary information, Fig. S2i). Notably, the changes in p-HSL and ATGL protein abundance and NEFA release, upon treatment with NTS, were absent in adipocytes in *Ntsr2* AKO mice (Supplementary information, Fig. S2j, k). We then explored the status of energy metabolism in these mice. Similar to *Ntsr2* BKO mice, *Ntsr2* AKO mice also presented a trend of increased thermogenesis (Supplementary information, Fig. S3a, b). It is widely accepted that the lipolysis of WATs is a major source of energy expenditure for non-shivering thermogenesis. On one hand, the elevation of lipolysis in the WAT of *Ntsr2* AKO mice may coordinate with the enhancement of thermogenesis in BATs. On the other hand, physiological factors other than lipolysis seem responsible for the elevation of body weight in *Ntsr2* AKO mice.

**Fig. 2:**
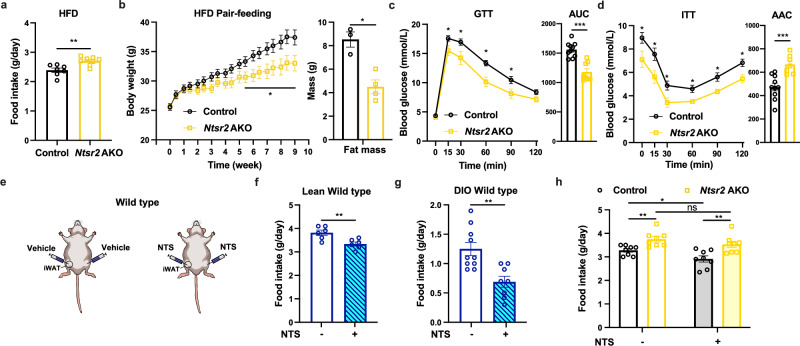
NTS-NTSR2 signaling in the adipose tissue regulated food intake. **a** Food intake of control and *Ntsr2* AKO mice (*n* = 7–8). **b**–**d** Changes of body weight (**b**), GTT (**c**) and ITT (**d**) of mice pair-fed by HFD (*n* = 6–10). **e** Schematic diagram of local NTS treatment. **f**, **g** Food intake of WT lean (**f**) or obese (**g**) mice upon NTS treatment (*n* = 6–12). **h** Food intake of control and *Ntsr2* AKO mice upon NTS treatment (*n* = 7–8). *P < 0.05; **P < 0.01; ***P < 0.001; ns, not significant.

In contrast to *Ntsr2* BKO mice, the food intake of *Ntsr2* AKO mice was remarkably increased (Fig. 2a; Supplementary information, Fig. S3c). Notably, a similar increase in food intake was also observed in *Ntsr2* AKO mice kept in the thermoneutral condition (Supplementary information, Fig. S3d). The abundance of NTS peptide in BATs and WATs was almost unchanged upon fasting-refeeding (Supplementary information, Fig. S3e). Then *Ntsr2* AKO mice were treated by pair feeding, a method to normalize the food intake of control and *Ntsr2* AKO mice and validate its significance for the physiological changes. If *Ntsr2* AKO mice on pair feeding presented comparable or even lower body weight than the control mice, it suggested that the change in food intake was the main reason for the differences in body weight. Notably, *Ntsr2* AKO mice with pair feeding of HFD showed lower body weight as well as fat mass weight (Fig. 2b). The pair-fed *Ntsr2* AKO mice also had a better performance in the GTT (Fig. 2c) and ITT (Fig. 2d). The contradictory results from either the ad-lib or pair-feeding experiments indicated that the metabolic changes in *Ntsr2* AKO mice were majorly induced by the food intake.

Based on the data related to the changes in food intake in *Ntsr2* AKO mice, we tested the local effects of recombinant NTS on WATs in vivo with a hydrogel containing recombinant NTS peptide (Supplementary information, Fig. S3f). The hydrogel was injected into the iWATs or eWATs of wild-type (WT) C57BL6/J mice (Fig. 2e; Supplementary information, Fig. S3g). Compared to the treatment of the hydrogel itself, the local release of NTS in WATs led to a decrease in food intake in both lean (Fig. 2f; Supplementary information, Fig. S3h) and obese mice (Fig. 2g). The treatment of NTS also led to downregulation of body weight and fat mass in the obese mice (Supplementary information, Fig. S3i, j). By contrast, the local release of NTS had no effects on the food intake of *Ntsr2* AKO mice (Fig. 2h), demonstrating that NTS regulated the food intake via the interaction with NTSR2 in adipocytes. However, the depletion of *Nts* specifically in the LECs (*Prox1-cre/ERT2::Nts*^*flox/flox*^, referred to as *Nts* KO),^25^ which presented high KO efficiency in the eWATs, had no effects on food intake (Supplementary information, Fig. S3k, l). These results suggested that the NTS-NTSR2 signaling regulated the food intake via a direct effect on the adipose tissue.

### NTS-NTSR2 signaling affects the metabolism of ceramide in adipocytes

**Fig. 3:**
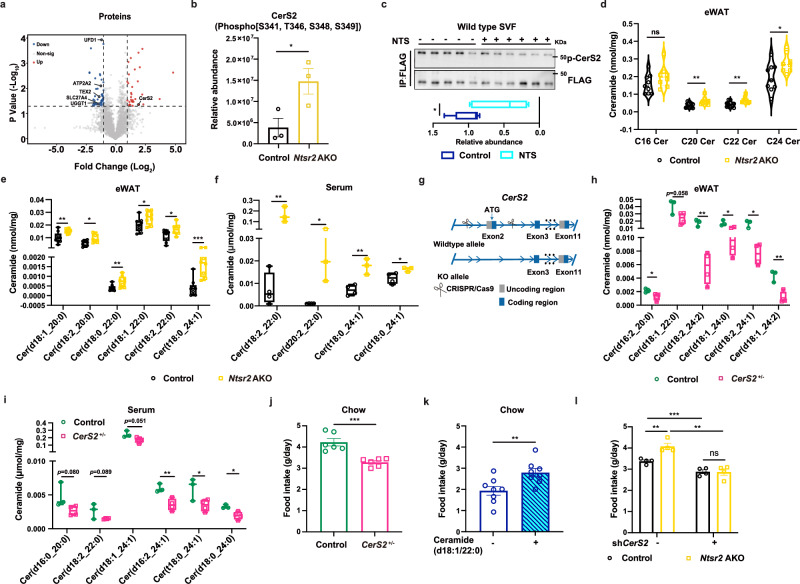
NTS-NTSR2 signaling regulated ceramide metabolism in WATs. **a** A volcano plot of phospho-proteomics data. **b** Relative abundance of p-CerS2 (*n* = 3). **c** p-CerS2 levels detected by pull-down and western blot analysis in the primary adipocytes (*n* = 5–6). **d**, **e** Ceramide C16-C24 levels (**d**) or individual ceramide levels (**e**) in eWATs of control and *Ntsr2* AKO mice (*n* = 6). **f** Individual ceramide levels in the serum of control and *Ntsr2* AKO mice (*n* = 6). **g** Scheme of the establishment of *CerS2*^+/–^ mouse strain. **h**, **i** Individual ceramide levels in the eWAT (**h**) and serum (**i**) of control and *CerS2*^+/–^ mice (*n* = 3). **j**, **k** Food intake of control and *CerS2*^+/–^ mice (**j**), and vehicle vs ceramide C22-treated mice (**k**); *n* = 6. **l** Food intake of control and *Ntsr2* AKO mice with *CerS2* knockdown (*n* = 4). *P < 0.05; **P < 0.01; ***P < 0.001; ns, not significant.

We then explored the mechanism by which the NTS-NTSR2 signaling in adipose tissues regulate food intake. We have reported that the NTS-NTSR2 signaling enhanced the phosphorylation of ERK (p-ERK) in BATs, but the depletion of *Ntsr2* in eWATs had no significant effects on the level of p-ERK (Supplementary information, Fig. S4a). The phospho-proteomics analysis on the eWATs from lean control and *Ntsr2* AKO mice with comparable body weight identified significant changes in the phosphorylation of multiple proteins (Fig. 3a; Supplementary information, Table S1). We observed the downregulation of the phosphorylation for multiple proteins related to endoplasmic reticulum (ER) stress and UPR (Supplementary information, Fig. S4b). It has been reported that C20–C24 ceramides, as the specific product of CerS2, can regulate the level of UPR as an upstream factor.^34,52^ Interestingly, *Ntsr2* depletion induced the elevation of phosphorylation in CerS2 protein (Fig. 3b). Other ceramide synthetases were not detected in the phospho-proteomics analysis, though it is still possible that NTSR2 controls the phosphorylation of the ceramide synthetases other than CerS2. We therefore hypothesized that the NTS-NTSR2 signaling regulates the phosphorylation of CerS2 to control the level of UPR.

As we did not manage to detect the peptide containing all the phosphorylation sites of CerS2 in iWATs with the phosphor-proteomics assay, we further confirmed the regulation of phosphorylated-CerS2 (p-CerS2) by NTS-NTSR2 signaling through overexpressing *CerS2* with a Flag-HA tag in the N-terminus in the SVF-derived adipocytes from iWATs (Supplementary information, Fig. S4c, d). By pulling down CerS2 with the Flag tag and performing immunoblot with a pan phosphor-Ser/Thr antibody, we identified that NTS suppressed the phosphorylation of CerS2 in the primary adipocytes (Fig. 3c). By contrast, the inhibitor of CK2,^29^ which has been identified as a kinase for the phosphorylation of CerS2 in 293 T cells, had no obvious effects on the phosphorylation of CerS2 (Supplementary information, Fig. S4e). The molecular weight difference between the native CerS2 and p-CerS2 bands was potentially due to glycosylation, which was consistent with a previous report.^29^

Ceramide is composed of one chain with stable length with hydroxyl groups and the other chain with variable length (the acyl chain synthesized by the CerS). The individual ceramide is usually named as Cer (d/t (two or three hydroxyl groups) XX (length of stable chain):X (number of double bonds)_XX (length of acyl chain):X (number of double bonds)). For example, Cer (d18:0_22:1) means ceramide with 18 carbons, 2 hydroxyl groups and zero double bond for the stable chain and 22 carbons and 1 double bond for the acyl chain. To investigate the abundance of ceramide with a certain length of acyl chain as a whole, we usually combined the ceramide with the same length of acyl chains and named them CXX (length of acyl chain) Cer. With the untargeted lipidomics assay, we observed the increase in C20–C24 ceramides as the products of CerS2, among all the ceramides (C16–C24 cereramides), in eWATs of *Ntsr2* AKO mice (Supplementary information, Fig. S4f and Table S2). Bn contrast, we did not observe changes in sphingosine or sphingomyelin (Supplementary information, Fig. S4g). These results were further validated on iWATs and eWATs by the targeted lipidomics assay, which was specifically designed for the detection of ceramide with various lengths of acyl chains^32^ (Fig. 3d; Supplementary information, Fig. S4h and Table S3; the technical details of targeted lipidomics were described in “Materials and Methods”). By contrast, there was no change in the abundance of ceramide in the BATs of *Ntsr2* AKO mice (Supplementary information, Fig. S4i). The top-5 differentially detected ceramides from eWATs were presented in Fig. 3e. NTS treatment on the primary adipocytes for 4 or 24 h decreased the abundance of C20–C24 ceramides, though different time courses of NTS treatment had specific effects on the abundance of ceramide with various lengths of acyl chain (Supplementary information, Fig. S4j). The hydrogel-embedded NTS treatment on the iWATs increased the abundance of C20–C24 ceramides (Supplementary information, Fig. S4k), but it had no effects on BATs (Supplementary information, Fig. S4l). Although the total concentration of C20–C24 ceramides was not dramatically changed in the serum, many C20–C24 ceramides were observed among the top differentially detected ceramides (Fig. 3f; Supplementary information, Fig. S4m and Table S4). In the phospho-proteomics analysis, we also discovered that the depletion of *Ntsr2* regulated the phosphorylation of ceramide transporter (CERT) (Supplementary information, Fig. S4n). As it has been reported that CERT was correlated to the abundance of C16 ceramide^53^ and its specificity for the length of acyl chain is still unresolved, it was unlikely to directly contribute to the effects of the NTS-NTSR2 signaling in the current context.

As the physiological functions of endogenous ceramide synthesis on food intake had not been reported, we tried to establish a mouse model with the deletion of *CerS2* gene. It has been reported that the homozygous depletion of *CerS2* was detrimental to the overall health of mice.^31^ In the current study, the depletion of one copy of *CerS2* gene (referred as *CerS2*^+/–^ mice, Fig. 3g; Supplementary information, Fig. S5a) led to a significant decrease in ceramide C24, trends of decreases in C20–C22, but without dramatic change in C16 in both eWATs and serum (Supplementary information, Fig. S5b, c and Tables S5, S6). The top-5 differentially detected ceramides in WATs and serum were presented at Fig. 3h, i. The expression of *CerS5* and *CerS6* was also unchanged in adipose tissues with the haploid depletion of *CerS2* (Supplementary information, Fig. S5d). By contrast, the liver of mice presented the significant decreases in individual ceramide C24, increases of individual C16 and upregulation of the expression of *CerS5* and *CerS6* (Supplementary information, Fig. S5e, f and Table S7), which was consistent with the literatures.^31,32^ The changes of C20–C24, but not C16, in WATs can be attributed to the differences of transcriptional regulation for *CerS5* and *CerS6* between the liver and WATs.

The *CerS2*^+/–^ mice showed a decrease in food intake (Fig. 3j). By contrast, the local treatment of ceramide C22, but not C16, in WATs upregulated the food intake of mice in vivo (Fig. 3k; Supplementary information, Fig. S5g). We treated the adipocytes for one hour and collected the samples immediately for lipidomics analysis. We observed a dramatic elevation of the intracellular C22 ceramide in the adipocytes with C22 ceramide treatment, which indicated that the long-chain ceramide may be internalized by the adipocytes (Supplementary information, Fig. S5h). Especially, *CerS2* knockdown via injecting adeno-associated virus (AAV) containing shRNA into iWATs or eWATs decreased the abundance of ceramides C20–C24 locally (Supplementary information, Fig. S5i–k and Table S8). *CerS2* knockdown also normalized the upregulation of food intake induced by *Ntsr2* AKO (Fig. 3i). These data indicated that the ceramide metabolism in the adipocytes contributed to the NTS-NTSR2 signaling-regulated food intake.

### NTS-NTSR2 signaling affects the level of UPR via ceramide metabolism

**Fig. 4:**
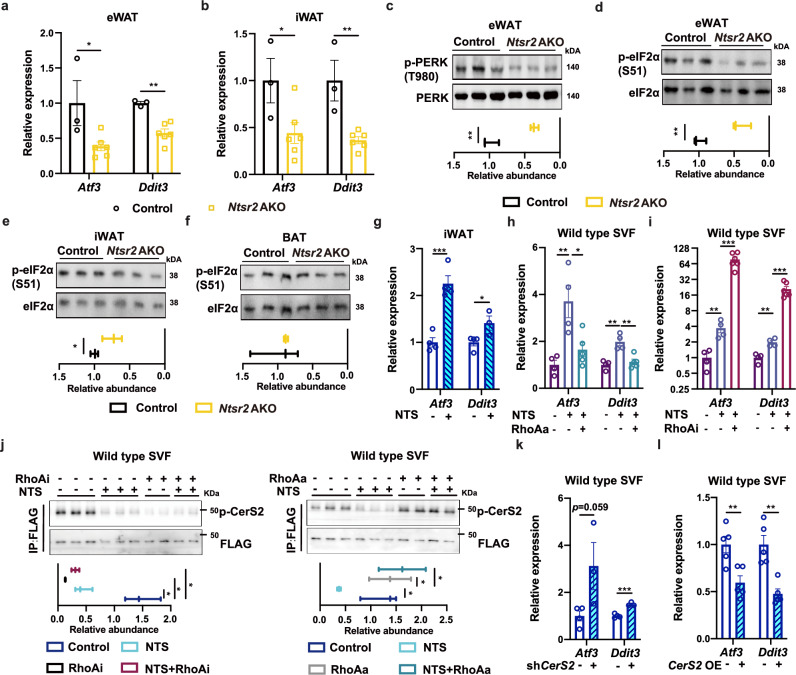
NTS-NTSR2 signaling regulated UPR via CerS2. **a**–**f** Expression levels of UPR-related genes (**a**, **b**) and proteins (**c**–**f**, *n* = 3) in the adipose tissues of control and *Ntsr2* AKO mice; *n* = 3–6. **g** Expression levels of UPR-related genes upon NTS treatment in vivo (*n* = 4). **h**, **i** Expression levels of UPR-related genes in the primary adipocytes upon the combinational treatment of NTS and RhoAa (**h**) or RhoAi (**i**); *n* = 4. **j** p-CerS2 levels in the primary adipocytes upon the combinational treatment of NTS and RhoAi or RhoAa (*n* = 2–3). **k**, **l** Expression levels of UPR-related genes in the primary adipocytes upon knockdown (**k**) or overexpression of *CerS2* (**l**); *n* = 4–5. *P < 0.05; **P < 0.01; ***P < 0.001.

As C20–C24 has been reported as a type of lipid to regulate UPR in cancer cells,^34,35^ we employed a collection of assays to test the changes of UPR-related factors in adipose tissues or primary adipocytes. Initially, we observed the downregulation of genes related to the UPR in WATs but not BATs of *Ntsr2* AKO (Fig. 4a, b; Supplementary information, Fig. S6a). Similar changes in UPR-related genes were observed in WATs of *Ntsr2* AKO mice upon pair-feeding (Supplementary information, Fig. S6b). *Ntsr2* AKO also downregulated the UPR-associated proteins, including phosphorylated PERK (p-PERK)^54,55^ and phosphorylated eIF2α (p-eIF2α), in WATs but not BATs (Fig. 4c–f). RNA-seq analysis of WATs further revealed that the depletion of *Ntsr2* downregulated the expression of genes related to UPR in WATs (Supplementary information, Fig. S6c and Table S9). By contrast, the phosphorylation of other integrated stress response factors, such as amino acid sensor GCN2^56–58^ and innate immunity response factor PKR,^59,60^ were not changed by *Ntsr2* AKO (Supplementary information, Fig. S6d, e). In addition, *Ntsr2* AKO did not change the expression of other upstream factors for UPR, including *Xbp1s* and *Atf6* (Supplementary information, Fig. S6f). Interestingly, treatment of NTS peptide increased the level of UPR-related factors in the fat in vivo as well as ex vivo (Fig. 4g; Supplementary information, Fig. S6g, h). However, the LEC-specific depletion of *Nts* had no effects on the expression of UPR-related genes in the fat in vivo (Supplementary information, Fig. S6i).

To further dissect the signaling between NTS-NTSR2 and UPR, we tested a collection of 13 chemicals that target various downstream factors of GPCR (Supplementary information, Table S10). Though most of the chemicals had no rational effects on the regulation of UPR-related genes by NTS, we did identify that RhoA was a potential factor linking the NTS-NTSR2 signaling and UPR in the primary adipocytes. At first, the treatment of RhoA antagonist (RhoAi) upregulated the expression of *Atf3* and *Ddit3* (Supplementary information, Fig. S6j), but the treatment of RhoA agonist (RhoAa) downregulated the expression of these two genes (Supplementary information, Fig. S6k). RhoAa prevented the NTS treatment-induced elevation of *Atf3* and *Ddit3* in the primary adipocytes (Fig. 4h), but RhoAi enhanced the effects of NTS (Fig. 4i). RhoAa but not RhoAi treatment also prevented the decrease of p-CerS2 induced by NTS treatment (Fig. 4j). These results together showed that the NTS-NTSR2 signaling regulated the function of CerS2 via RhoA.

Consistent with our hypothesis that CerS2 was a downstream factor of the NTS-NTSR2 signaling, we discovered that the knockdown or overexpression of *CerS2* (Supplementary information, Fig. S7a, b) controlled the expression of UPR-related factors (Fig. 4k, l; Supplementary information, Fig. S7c) in the primary adipocytes. Notably, the knocking-down of *CerS2* also increased the level of UPR-related factors in the primary adipocytes with *Ntsr2* depletion (Supplementary information, Fig. S7d) and prevented the NTS-induced elevation of UPR-related factors (Supplementary information, Fig. S7e). These data indicated that CerS2 is a downstream factor of NTS-NTSR2 signaling in regulating UPR. It has been reported that C16 ceramide upregulated the level of UPR in the hypothalamus.^61^ By contrast, we found that the treatment of C22 ceramide, but not C16, downregulated the expression of UPR-related factors in the primary adipocytes (Supplementary information, Fig. S7f–i). *CerS2*^+/–^ mice presented the elevation of UPR-related factors in WATs (Supplementary information, Fig. S7j, k), but the local treatment of ceramide C22 in vivo downregulated the level of UPR-related factors in WATs (Supplementary information, Fig. S7l). These results suggested that the NTS-NTSR2 signaling regulated the synthesis of ceramides C20–C24, which in turn affected the level of UPR in WATs.

### NTS-NTSR2 signaling affects food intake via the regulation of GDF15

**Fig. 5:**
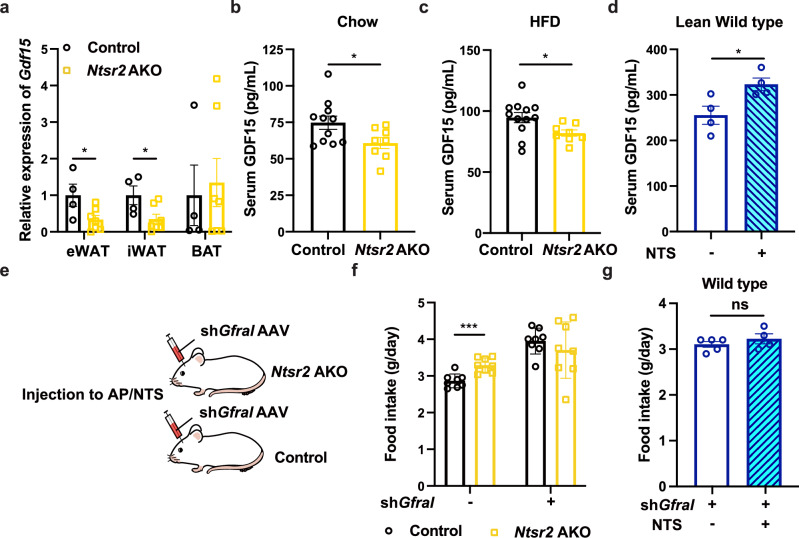
Decreasing GDF15 levels led to an increase in food intake in *Ntsr2* AKO mice. **a** Expression levels of *Gdf15* in the adipose tissues (*n* = 4–7). **b**–**d** GDF15 protein concentrations in the serum of control and *Ntsr2* AKO mice fed by a chow diet (**b**, *n* = 8–11), HFD (**c**, *n* = 8–12) or treated by NTS (**d**, *n* = 5). **e** Illustration of the experimental design. **f** Food intake of control and *Ntsr2* AKO mice with or without knockdown of *Gfral* (*n* = 8). **g** Food intake of mice treated by NTS in iWATs with *Gfral* knockdown (*n* = 5). *P < 0.05; ***P < 0.001; ns, not significant.

The data derived from the *Ntsr2* AKO mice and the literature^36^ inspired us to hypothesize that UPR, as a part of the integrative stress response (ISR), may regulate food intake via controlling the production of GDF15. Notably, we discovered that the *Gdf15* gene, and its encoding product GDF15, were downregulated in eWATs and iWATs (but not BATs) of *Ntsr2* AKO mice (Fig. 5a; Supplementary information, Fig. S8a). It has been reported that GDF15 may regulate the body weight of mice via muscle.^62^ Therefore, we measured the expression of *Gdf15* gene in the skeletal muscle of *Ntsr2* AKO mice. However, we did not observe such decreases in *Gdf15* gene expression in the skeletal muscle of *Ntsr2* AKO mice (Supplementary information, Fig. S8b). The GDF15 protein was also downregulated in the serum of *Ntsr2* AKO mice fed by either a chow diet (Fig. 5b) or HFD (Fig. 5c). The decreases of GDF15 in the serum of *Ntsr2* AKO mice were also identified in the thermoneutral condition (Supplementary information, Fig. S8c). By contrast, the local release of NTS peptide by hydrogel in iWATs upregulated the serum GDF15 in both lean and obese mice (Fig. 5d; Supplementary information, Fig. S8d).

To validate whether the food intake differences between control and *Ntsr2* AKO mice were dependent on GDF15 signaling, we knocked down GDF15’s receptor *Gfral*^63^ in the area postrema/nucleus of the solitary tract region of the brain via AAV injection (Fig. 5e). As the interaction between GDF15 and GFRAL was well-established, we used it as a tool to validate our hypothesis. The effects of knocking-down on the expression of *Gfral* in the area postrema/nucleus of the solitary tract region were validated by fluorescent in situ hybridization (FISH) (Supplementary information, Fig. S8e). The downregulation of *Gfral* also led to increases in food intake (Supplementary information, Fig. S8f), which was consistent with what has been reported. Notably, the changes in food intake induced by *Ntsr2* AKO or NTS local treatment in iWATs were abolished by knocking down *Gfral* (Fig. 5f, g). In comparison to the body weight of the mice before the AAV injection, *Gfral* knockdown in both control and *Ntsr2* AKO mice induced a lower speed of body weight increase of the *Ntsr2* AKO mice by normalizing the food intake (Supplementary information, Fig. S8g). The decreases in body weight elevation were due to the enhanced thermogenesis in the *Ntsr2* AKO mice. We also treated the mice with recombinant GDF15 via intraperitoneal injection. We found that by normalizing the serum concentration of GDF15 (Supplementary information, Fig. S8h), we managed to normalize the food intake of the control and *Ntsr2* AKO mice (Supplementary information, Fig. S8i). In addition, we tried to normalize the serum concentration of GDF15 by enhancing its endogenous production by treating the mice with PERK agonist (PERKa).^64^ Consistently, the PERKa treatment normalized the serum level of GDF15 as well as food intake in *Ntsr2* AKO mice (Supplementary information, Fig. S8j, k). These results indicated that NTS-NTSR2 signaling in WATs regulates food intake via GDF15 signaling. Notably, We recently have validated that the UPR-related factor PERK controlled the food intake as well as metabolic homeostasis in DIO mice via GDF15.^65^

**Fig. 6:**
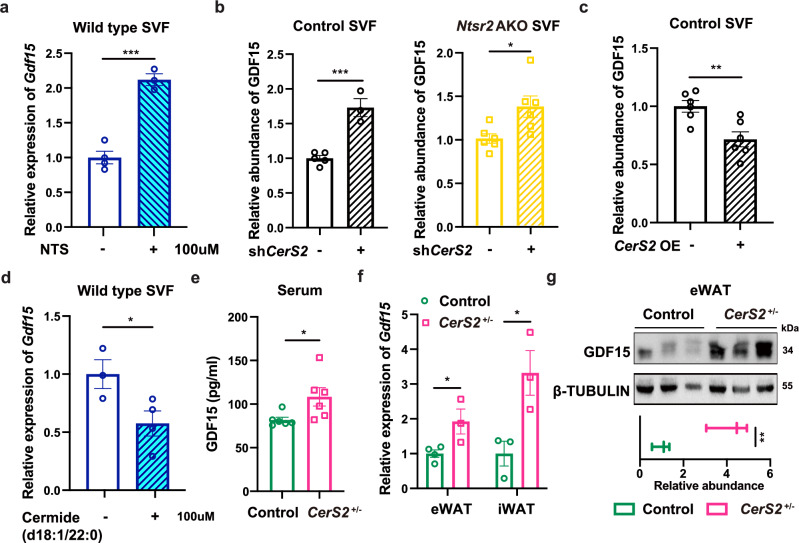
NTS-NTSR2 signaling regulated the production of GDF15 via CerS2. **a** Expression level of *Gdf15* upon NTS treatment in the primary adipocytes of WT mice (*n* = 3–4). **b**–**d** Expression levels of GDF15 protein upon *CerS2* knockdown (**b**, *n* = 3–5), *CerS2* overexpression (**c**, *n* = 6) and ceramide C22 treatment (**d**, *n* = 3–4) in primary adipocytes. **e**–**g** Serum concentrations of GDF15 (**e**, *n* = 6), mRNA expression levels of *Gdf15* (**f**, *n* = 3) and GDF15 protein abundance in adipose tissues (**g**, *n* = 3) of control and *CerS2*^+/–^ mice. *P < 0.05; **P < 0.01; ***P < 0.001.

We then tested whether the NTS-NTSR2 signaling regulated the production of GDF15 via controlling ceramide metabolism. The treatment of Rhoi upregulated the expression of *Gdf15* (Supplementary information, Fig. S9a), but the treatment of RhoAa downregulated the expression of *Gdf15* (Supplementary information, Fig. S9b). We found that NTS treatment upregulated the expression of *Gdf15* in the SVF-derived primary adipocytes compared to the vehicle treatment, but this phenotype was abolished in the cells with *CerS2* knockdown (Fig. 6a; Supplementary information, Fig. S9c) or with the treatment of RhoAa (Supplementary information, Fig. S9d). *CerS2* knockdown upregulated the production of GDF15 protein in both control and *Ntsr2* AKO primary adipocytes (Fig. 6b). By contrast, the overexpression of *CerS2* downregulated the production of GDF15 protein (Fig. 6c; Supplementary information, Fig. S9e) in both control and *Ntsr2* AKO primary adipocytes. In addition, the treatment of C22 ceramide, but not C16, indeed downregulated the expression of *Gdf15* in the primary adipocytes (Fig. 6d; Supplementary information, Fig. S9f). *CerS2*^+/–^ mice presented an elevation of serum GDF15 concentration (Fig. 6e), upregulation of *Gdf15* gene (Fig. 6f) and GDF15 protein (Fig. 6g) in WATs compared to the control mice. However, the treatment of ceramide C22 in vivo downregulated the expression of the *Gdf15* gene or GDF15 protein in WATs (Supplementary information, Fig. S9g) and GDF15 protein in serum (Supplementary information, Fig. S9h). In addition, *Cers2* knockdown in iWATs also led to an elevation of serum GDF15 levels (Supplementary information, Fig. S9i). Together, these data suggested that NTS-NTSR2 signaling regulated the C20–C24 ceramide synthesis, which affected the production of GDF15 and food intake in mice.

### The correlation between ceramides and food intake in humans

**Fig. 7:**
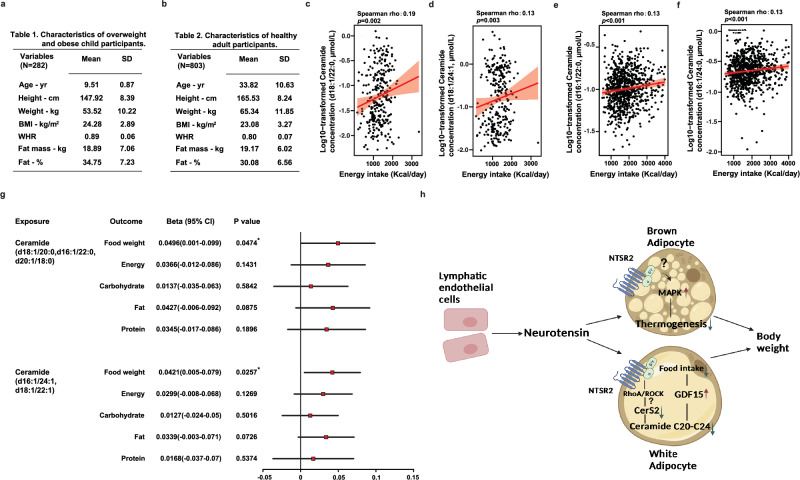
The correlation between ceramides C20–C24 and food intake in humans. **a**, **b** Basic information of child (**a**) and adult (**b**) participants in the study. **c**–**f** A positive correlation was obtained based on multivariable linear regression models between the serum concentration of ceramides C20–C24 and energy intake in the cohorts of children (**c**, **d**) and adults (**e**, **f**). **g** Summary of Mendelian randomization analysis in adults. **h** Illustration of the mechanism identified in this study.

To examine the generalization of our findings in humans, we collected the relevant information and analyzed the correlation between plasma ceramide C20–C24 levels and daily intake of food or energy in both children (282 participants) and adults (803 participants). The basic characteristics of these two populations are presented in Fig. 7a, b. A positive association between plasma levels of ceramides C20–C24 and energy intake was observed in both populations (Fig. 7c–f). Based on the results from a recent systemic analysis of the correlation between serum concentration of metabolites and food intake, the correlation identified in the current study was among the top tier.^66^ As we cannot confirm the correlation of ceramide in the paired WATs and serum due to the lack of samples, it is possible that the correlation between ceramide and food intake we observed here was due to tissues other than WATs in humans. Notably, a potential causal relation between plasma levels of ceramide C20–C24 and weight of daily food intake was observed using Mendelian randomization analysis based on the data from UK Biobank and METSIM study (Fig. 7g; Supplementary information, Tables S11, S12; see details of Mendelian randomization analysis in “Materials and Methods”). In addition, the serum C20–C24 ceramide levels were positively correlated to body weight, fat mass, body mass index, blood glucose level, serum triglyceride level, serum total cholesterol level, and serum low-density lipoprotein cholesterol for the adults (Supplementary information, Fig. S10), though the correlations between C20–C24 ceramides and these physiological parameters were much weaker for the children (Supplementary information, Fig. S11). These results suggested that the ceramide metabolism axis may also be of significance in the regulation of food intake as well as metabolic homeostasis in humans.

## Discussion

NTS has been recognized as an important peptide in the regulation of metabolic homeostasis for decades, though most studies on the functions and underlying mechanism of NTS were performed in the CNS or intestine. Our previous study has revealed the anti-thermogenic effects of LEC-produced NTS via its interaction with NTSR2 in BATs through paracrine signaling. In the current study, we validated this thermogenic phenotype and further identified that the NTS-NTSR2 signaling in WATs regulated food intake via its impact on ceramide metabolism. NTS-NTSR2 signaling affected the synthesis of ceramides C20–C24, which induced the changes in GDF15 and food intake. These results indicated that the NTS-NTSR2 signaling in the peripheral tissues may play a key role in keeping the balance between BAT-dependent thermogenesis and WAT-dependent food intake. As the NTS-NTSR2 signaling can suppress thermogenesis in the BAT and suppress food intake in the WAT simultaneously, it is an important factor in maintaining metabolic homeostasis. The disruption of NTS-NTSR2 signaling in either of the tissues may lead to abnormal elevation of body weight as well as metabolic disorders (Fig. 7h).

As the Adipoq-cre targets both WATs and BATs, we observed the elevation of energy expenditure in both *Ntsr2* BKO and AKO mice. These results indicate that the NTS-NTSR2 signaling has similar effects on BATs in these two strains. However, we observed the distinct phenotypes of food intake and body weight between *Ntsr2* BKO and AKO mice, which is likely due to the different effects of NTS-NTSR2 signaling on different cell types. NTS-NTSR2 signaling controls MEK-ERK signaling in BATs but UPR in WATs. This common feature of GPCR highlights the significance of studying the NTS-NTSR2 signaling in various cell types specifically.

In the previous study, we performed an AAV injection-based experiment to validate the anti-thermogenic effects of NTS in BATs. Because AAV-mediated knockdown may disappear very soon, it is difficult to understand the long-term physiological effects of this model. In addition, the knockdown of *Ntsr2* is not cell-type specific. In the current study, we used the *Ntsr2* BKO model to confirm the long-term physiological function for the suppression of NTSR2 specifically in the BAT, which has not been tested in the previous study.

The fact that the depletion of *Nts* in LECs had no effects on food intake indicated potential existence of endogenous ligands for NTSR2 other than NTS. Actually, it has been demonstrated in the literature that glycine can activate NTSR2 with PRESTO-Salsa-based GPCR reporter system.^67^ The details about how the non-canonical ligand for NTSR2 regulated metabolic homeostasis can be studied in the future.

NTS can interact with both NTSR1 and NTSR2, but the downstream signaling pathways of these two GPCRs are strikingly different. Unlike NTSR1,^68–72^ the effects of NTS stimulation on NTSR2 are controversial. For mouse NTSR2, NTS induced coupling of G_q_ to NTSR2 in a system of *Xenopus Laevis* oocytes but not HEK293 cells or COS cells.^73–76^ For human NTSR2, NTS behaved as a neutral antagonist in COS cells and HEK293 cells but an activator of MEK-ERK signaling in CHO cells.^77–80^ Previously, we have reported that NTS controlled the MEK-ERK signaling in BATs. However, the results of the current study indicated that the MEK-ERK signaling was not affected by the depletion of *Ntsr2* in WATs. The tissue- and context-specificity are the outstanding features of the function of GPCR.^81^ These studies with contradictory conclusions highlighted the importance of studying NTS-NTSR2 signaling with cell type-specific models in physiological conditions.

In an ex vivo model, we have previously identified that NTS activates MEK-ERK signaling which is responsible for its anti-thermogenic effects.^23^ In the current study, we discovered that NTS may exert inhibitory effects on food intake via NTSR2 in WATs, but not BATs, via regulating the RhoA-CERS2-GDF15 axis in vivo. As far as we know, this is the first evidence to support the hypothesis that NTS may exert neurological regulation via direct interactions with peripheral tissues. Further studies are required to elucidate whether it is a common mechanism or an adipocyte-specific phenomenon.

Previously, we have reported that treatment with the NTSR2 antagonist NTRC-824 enhanced thermogenesis without obvious effects on food intake in mice.^23^ Encouragingly, the *Ntsr2* BKO and AKO mice in the current study also presented enhanced thermogenesis. The inconsistencies in the food intake phenotypes between *Ntsr2* AKO mice and mice treated with NTRC-824 may be due to several differences between the two studies: a) NTRC-824 treatment exerted its effects on the whole body in comparison to adipocyte-specific depletion of *Ntsr2* shown in the current study. The complicated effects of NTSR2 inhibition on various cell types may disturb the feeding behavior of mice. b) NTRC-824 treatment was performed in obese mice while *Ntsr2* depletion was carried out when mice were still lean. The dramatic difference between lean and obese mice may affect the phenotypes associated with the feeding behavior. c) Many chemicals have more than one target in vivo. Similarly, NTRC-824 may have unknown off-target effects other than inhibiting the activity of NTSR2.

Ceramide has been broadly recognized as a type of lipid with important bioactivities. Ceramide may exert diverse effects on metabolism-related phenotypes via interaction with brown/beige adipocytes or the CNS.^61,82,83^ Many groups, including ourselves, have demonstrated that the total ceramide is an important factor in senescence or aging.^84,85^ Notably, it has been reported that the ICV administration of mixed species of ceramide to rainbow trout induced decreases of food intake in fish.^86^ By contrast, the effects of ceramide on the food intake of mammals were complicated. ICV injection or local ablation of ceramide C6 in hypothalamus of mice may either increase food intake^87^ or have no effects on food intake.^83^ The current studies identified that the ceramide C20–C24, as the specific product of CerS2 from peripheral tissues, may regulate the food intake in both mice and humans. The metabolism of ceramide has the potential to become a therapeutic target for obesity.

The UPR is an important pathway for the regulation of ER stress. It has been reported in several studies that the intracellular abundance of ceramide was associated with ER stress.^88^ In the macrophage, the ASMase can induce ER stress via elevation of ceramide and oxidized-LDL.^89^ In the CNS, treatment of ceramide synthesis inhibitor myriocin or depletion of *CerS6* suppressed the ER stress in the hypothalamus.^61,90^ Especially, it has been discovered that dihydroceramide can bind directly to ATF6, a key regulator of UPR, which activates the downstream signaling.^91^ We therefore hypothesized that C20–C24 ceramides may specifically interact with a protein involved in the regulation of PERK. This interaction controlled the phosphorylation of PERK, the production of GDF15 and the food intake. As the current study is a physiological study in general, this candidate protein interacting with C20–C24 ceramides awaits to be discovered by biophysical/chemical biology studies in a separate project.

In summary, our study provided an inspiring insight into the function and relevant mechanism of NTS-NTSR2 signaling in the context of metabolic regulation. In addition to NTS, other secretory proteins produced by LECs have been discovered.^92–96^ Together, these studies will inspire future work on how the LEC interacts with peripheral organs/tissues via secretory proteins, as well as how it regulates metabolic homeostasis.

## Materials and methods

### Ethical declaration

The ceramides and food intake correlation study in children has been approved by the ethics committee of Peking University Third Hospital (ethnic approval number 2021-283-06). The ceramides and food intake correlation study in adult has been approved by the Ethics Committees of Fudan University (FE20064) and Zhongshan Hospital (B2019-089R). The informed consents to participate in the study have been obtained from either the adult participants or the parents or legal guardian of children under age 16.

### Reagents

The information on key reagents involved in this study was presented in Supplementary information, Table S13.

### Mouse models

All animal experiments were performed according to procedures approved by the Animal Ethics Committee of Fudan University. Mice were maintained under a 12-h light/12-h dark cycle at constant temperature (23 °C) with free access to food and water. Each mouse was kept individually in the cage to measure food intake. Food intake was measured based on the differences in the amount of diet that we provided in the morning on the first day and the amount of diet left in the morning of the second day. The diet was replaced every day to keep the measurement of food intake accurate. For each mouse, we measured the food intake for a week and calculated the average food intake for this mouse. Then the data were summarized as the food intake for certain groups of mice. WT C57B6/J mice, *Ntsr2*^*flox/flox*^ mice, *Nts*^*flox/flox*^ and *Cers2*^+/–^ mice were obtained from GenPharmatech. The *Ucp1-cre*, *Adipoq-cre* and *Prox1-cre/ERT2* mice were obtained from the Jackson Lab.

To generate *Ntsr2* BKO mice, *Ntsr2*^*flox/flox*^ mice were crossed with *Ucp1-Cre* mice. To obtain *Ntsr2* AKO mice, *Ntsr2*^*flox/flox*^ mice were crossed with *Adipoq-Cre* mice. Mouse genotyping was performed using the Mouse Direct PCR Kit (Cat# B40015, Bimake). The genotyping primers used were as follows: *Ntsr2 flox*: 5’-GCAAAGCTGCTTCTCTTTACTGAG-3’; 5’-AGATAGATGGACCTCAAAGGCAG-3’; *Adipoq-cre*: 5’-ACGGACAGAAGCATTTTCCA-3’; 5’-GGATGTGCCATGTGAGTCTG-3’; *Ucp1-Cre*: 5’-GTCCTGGAACGTCATCATGTTTG-3’；5’-GCTTCCTTCACGACATTCAACAG-3’; *Cers2*^+/–^: 5’-TTCATCCACAAGAGCAGTGACCAG-3’; 5’-AAGTCCTCACCTTCAAAGCAAGC-3’. The pair-feeding experiment in a staggering manner was performed according to the published protocols.^97,98^ For studies of WT obese animals, 8-week-old male mice were fed by HFD for 8 weeks before the experiments started.

### Stereotaxic surgery, virus injections and FISH

Male mice aged 10–12 weeks were anesthetized with isoflurane (3%–4% isoflurane for induction and 1%–2% isoflurane for maintenance) by intraperitoneal injection and then mounted in a stereotaxic apparatus (RWD, Model 68045). An incision was made to expose the skull and a small craniotomy was performed for bilateral injections with the following parameters: posterior bregma –7.65 mm; 0.00 mm lateral to bregma, depth at 4.9 mm from the skull. 0.3 µL of AAV was injected on each side with a micro-syringe pump over 10 min. AAV (30 nL, WZ Biosciences) was injected slowly with a micro-syringe pump over 10 min. To enable recovery and AAV expression, mice were housed for a minimum of 7 days following virus injection before the experiments started. The sequences for shRNA were provided in Supplementary information, Table S14.

In situ hybridization of RNA molecules was achieved by using amplification-based single molecule in situ hybridization (asmFISH) method as described in our previous study.^99^ SEERNA® ISH RNA Fluorescence in Situ Detection Kit (SEERNA Bioscience) was used under the guidance of standard protocol of the manual. The label probe was conjugated to Alexa fluor 488, Cy3, Texas Red, Cy5 or Alexa fluor 750 (ThermoFisher Scientific).

After OCT-embedding, tissue sections in 10-μm thickness were first fixed in 4% (w/v) paraformaldehyde (Sigma) for 5 min, and washed twice with diethyl pyrocarbonate (DEPC)-treated PBS, followed by dehydration in an ethanol series. After three washes with Wash Buffer (SEERNA Bioscience), sections were permeabilized with 0.1 M HCl at 37 °C for 5 min.

The sections were then incubated in order with the following solutions: target probes in hybridization mix at 37 °C for 4 h, ligation mix at 37 °C for 30 min, splint primers in the circularization mix at 37 °C for 30 min, and amplification mix for rolling circle amplification at 30 °C overnight. Slides were washed with Wash Buffer (SEERNA Bioscience) three times at room temperature (RT) after each step. After that, the sections were incubated in hybridization buffer containing the label probes at RT for 30 min. Finally, the slides were mounted with SlowFade Gold Antifade Mountant (ThermoFisher Scientific) medium containing 0.5 μg/mL DAPI (Sigma), which were then ready for image acquisition using the Leica DM6B microscope equipped with a DFC9000GT camera using the 40× or 20× objective lens. The targeting regions of the probes were provided in Supplementary information, Table S14.

### Pharmacological treatment

Ten-week-old male mice received intraperitoneal injection of GDF15 recombinant protein (0.2 mg/kg) (Cat# ab202199, Abcam) or PBS with free access to a chow diet for 10 days. Various assays were performed during the treatment. At the end of the experiment, the serum, fat pads, and liver were collected and stored for later analysis.

### Body composition, fecal energy, and indirect calorimetry

Body composition to determine lean mass and fat mass values were obtained before the experiment with a mini-Spec LF50 (Bruker). The energy expenditure was measured by the Promethion CLAMS (Sable Systems International) housed within a temperature-controlled environmental chamber at Fudan University. Before the experiment started, the mice were housed in the CLAMS for 48 h. The data were processed as described before.^23^ The energy content of diet and feces was measured by a bomb calorimeter (IKA) according to the manufacturer’s guidelines.

### Local release of NTS

The hydrogel was synthesized and the NTS peptide was loaded into the hydrogel as described before.^100,101^ In brief, the sodium hyaluronate, poloxamer 407 and ultrapure water were mixed, centrifuged and stored in the fridge. The NTS peptide was added to the hydrogel at 1.5 mg/mL. For the treatment, 100 µL of hydrogel with or without NTS was injected into iWATs once every two days. The tissues and serum were collected from mice with treatment for 6 days.

### Culture and differentiation of mouse pre-adipocytes

Inguinal adipose tissues were dissected from 7–10-week-old C57/Bl6J male mice, minced, and digested in PBS with 10 mg/mL BSA (Cat# A600332-0100, BBI Life Sciences) and 10 mg/mL collagenase (Cat# 11088858001, Roche) for 60 min at 37 °C. The digested solution was filtered through a 100-µm cell strainer, centrifuged at 800 rpm for 5 min and the red blood cells were lysed by ACK buffer (Cat# A10492-01, Gibco). The SVF pellet was then suspended in the DMEM medium with 10% FBS and 1% P/S for cell culture experiments. The SVF cells were differentiated into adipocytes with a growth medium containing 5 µg/mL insulin, 0.5 mM isobutylmethylxanthine, 1 µM rosiglitazone, 1 µM dexamethasone for two days initially. Then we refed the cells with a new growth medium containing 5 µg/mL insulin for another two days. Afterward, the cells were maintained in a growth medium without insulin. Cells were fully differentiated after 6–8 days.^102^ As the expression of *Ntsr2* in the differentiated adipocytes was not detectable, we overexpressed *Ntsr2* in the adipocytes via AAV infection. The sequences for shRNAs were provided in Supplementary information, Table S14.

### RNA Isolation/qRT-PCR

TRIzol (Cat# 15596018, ThermoFisher Scientific) was used for RNA isolation. Extracted RNA (500 ng) was converted into cDNA using the PrimeScript™ RT reagent Kit (Cat# RR037A, Takara). qRT-PCR was performed using an Applied Biosystems QuantStudio 5 and TB Green Premix Ex Taq (Cat# RR420B, Takara). Fold change was determined by comparing target gene expression with the reference gene *36b4*. Primers are listed in Supplementary information, Table S14.

### RNA-seq

Total RNA was extracted from adipose tissues of mice fed by chow diet as described before.^103^ Sequencing libraries were generated using NEBNext® UltraTM RNA Library Prep Kit for Illumina® (NEB, USA) and index codes were added to attribute sequences to each sample. Briefly, mRNA was purified from total RNA using poly-T oligo-attached magnetic beads. Fragmentation was carried out using divalent cations under elevated temperature in NEBNext First Strand Synthesis Reaction Buffer (5×). First-strand cDNA was synthesized using random hexamer primers and M-MuLV Reverse Transcriptase (RNase H–). Second-strand cDNA synthesis was subsequently performed using DNA Polymerase I and RNase H. Remaining overhangs were converted into blunt ends via exonuclease/polymerase activities. After adenylation of 3’-ends of DNA fragments, NEBNext Adaptors with hairpin loop structures were ligated to prepare for hybridization. To select cDNA fragments with 250–300 bp, the library fragments were purified using the AMPure XP system (Beckman Coulter). Then 3 µL of USER Enzyme (NEB) was used with size-selected, adaptor-ligated cDNA at 37 °C for 15 min, followed by 5 min at 95 °C before PCR. Then PCR was performed with Phusion High-Fidelity DNA polymerase, Universal PCR primers, and Index Primer. At last, PCR products were purified (AMPure XP system) and library quality was assessed on the Agilent Bioanalyzer 2100 system. The clustering of the index-coded samples was performed on a cBot Cluster Generation System using TruSeq PE Cluster Kit v3-cBot-HS (Illumia). After cluster generation, the library preparations were sequenced on an Illumina Hiseq platform and 125–150 bp paired-end reads were generated.

Raw data of fastq format were firstly processed through in-house perl scripts. In this step, clean data were obtained by removing reads containing adapter and trimming low-quality base with Trimmomatic.^104^ Index of the reference genome was built using Hisat2 and paired-end clean reads were aligned to the reference genome using Hisat2. StringTie was applied to the bam file for expression quantification and the RNA-seq count data were obtained. Finally, differential expression analysis of two groups was performed using the DESeq2 R package.^105^ The raw data were deposited to GEO with the accession number GSE274164.

### Adipocyte-specific transcriptomic analysis by TRAP/RNA-seq

Ribosome pulldown was conducted as previously described,^50^ with modifications. Frozen adipose tissues were lysed using Dounce homogenizers in lysis buffer (20 mM HEPES pH 7.5, 15 mM MgCl_2_, 150 mM KCl, 1% NP-40, 100 µg/mL cycloheximide, 1 mM DTT, 0.1 U/μL RNase inhibitor, and 1× Halt EDTA-free protease inhibitor) and incubated on ice for 15 min with vortexing every 5 min. After lysates were centrifuged at 13,000 rpm for 10 min at 4 °C, the infranatant layer below the top lipid layer was collected. Immunoprecipitation was conducted by rotating the lysates incubated with GFP antibody (Cat# ab290, Abcam) for 1 h at 4 °C and subsequently with Dynabeads Protein G (Cat# 10003D, ThermoFisher Scientific) for 30 min at 4 °C. After washing three times using wash buffer (20 mM HEPES pH 7.5, 15 mM MgCl_2_, 350 mM KCl, 1% NP-40, and 100 µg/mL cycloheximide), RNA was extracted from the immunoprecipitates using a Qiagen RNeasy Micro kit and purified by on-column DNA digestion using a Qiagen RNase-Free DNase Set according to the manufacturer’s instructions. For RNA-seq library construction, the purified RNA (100 ng) was processed by an NEBNext rRNA Depletion Kit (Cat# E7400L, New England BioLabs) for removal of ribosomal RNA, and first-strand cDNA was synthesized by Maxima Reverse Transcriptase (Cat# EP0753, ThermoFisher Scientific). Double-stranded cDNA was generated from the first-strand cDNA by an NEBNext mRNA Second Strand Synthesis kit (E6111S), and sequencing libraries were generated by tagmentation using Nextera XT DNA Library Preparation kit. The libraries were quantified by Qubit, analyzed by Agilent Bioanalyzer, and sequenced on an Illumina NovaSeq6500. The sequencing reads were pre-processed by FastQC (http://www.bioinformatics.babraham.ac.uk/projects/fastqc) for quality control and then aligned to the mm10 mouse reference genome using STAR.^106^ Low-quality mapped reads including duplicates were determined and excluded by bamUtils,^107^ and filtered reads were assigned to transcriptome and quantified by featureCounts.^108^ Differential expression analysis of two groups was performed using edgeR^109^ after lowly expressed genes (CPM < 1 in at least 20 different samples among 24) were filtered out.

### Phosphoproteomics

#### Trypsin digestion

The FASP digestion was adapted for the following procedures in Microcon PL-10 filters as previously described.^110^ After three-time buffer replacement with 8 M Urea and 100 mM Tris-HCl, pH 8.0, proteins were reduced by 10 mM DTT at 37 °C for 30 min, and followed by alkylation using 30 mM iodoacetamide at 25 °C for 45 min in the dark. Then the sample was washed three times with digestion buffer (30 mM Tris-HCl, pH 8.0), and the digestion was carried out with trypsin (enzyme/protein as 1:50) at 37 °C for 12 h. After digestion, the solution was filtrated out and the filter was washed twice with 15% ACN, and all the filtrates were pooled and vacuum-dried.

#### Phosphopeptide enrichment

The home-made TiO_2_ microcolumns (peptide:TiO_2_ = 1:10) were washed by 100% ACN. Peptides were dissolved with loading buffer (1 M glycolic acid, 80% ACN and 5% TFA). After loading peptides onto the microcolumn twice, the microcolumns were washed twice with loading buffer and then with washing buffer (80% ACN and 1% TFA) twice. The phosphopeptides were successively eluted by 2 M ammonia hydroxide and then 1 M ammonia hydroxide with 30% ACN. The elutes were vacuum-dried by SpeedVac.

#### NanoLiquid chromatography-mass spectrometry (NanoLC-MS) analysis

NanoLC-MS/MS analysis was performed using an EASY-nLC 1200 system (ThermoFisher Scientific) coupled to an Orbitrap Fusion Lumos mass spectrometer (ThermoFisher Scientific). A one-column system was adopted for all analyses. Samples were analyzed on a home-made C18 analytical column (75 µm i.d. × 25 cm, ReproSil-Pur 120 C18-AQ, 1.9 µm (Dr. Maisch GmbH, Germany)).^111^ The mobile phases consisted of Solution A (0.1% formic acid) and Solution B (0.1% formic acid in 80% ACN). The peptides for LFQ analysis were eluted using the following gradients: 2%–5% B in 2 min, 5%–35% B in 100 min, 35%–44% B in 6 min, 44%–100% B in 2 min, 100% B for 10 min, at a flow rate of 200 nL/min. The phosphopeptides were eluted using the following gradients: 2%–5% B in 3 min, 5%–35% B in 40 min, 35%–44% B in 5 min, 44%–100% B in 2 min, 100% B for 10 min, 100%–5% B in 2 min, 5% B in 3 min, 5%–100% B in 2 min, 100% B for 10 min at a flow rate of 200 nL/ min. Data acquisition mode was set to obtain one MS scan followed by HCD-MS/MS acquisitions with a cycle time of 2 s. The normalized collision energy (NCE) was set as 30.

#### Raw data processing

The results were processed with UniProt human protein database (75,004 entries) and using Protein Discoverer (Version 2.4, ThermoFisher Scientific) with Mascot (Version 2.7.0, Matrix Science).^112^ The mass tolerances were 10 ppm for precursor and fragment Mass Tolerance 0.05 Da. Up to two missed cleavages were allowed. Carbamidomethylation on cysteine was set as a fixed modification, and acetylation on the protein N-terminus and oxidation of methionine were set as variable modifications. For phosphopeptides, additional parameters were set: the phosphorylation on serine, threonine and tyrosine as variable modifications.

### RNA-seq data analyses

The in-house perl scripts were applied to process raw data in fastq format. In order to obtain clean data, reads containing adapters were removed and low-quality bases were trimmed with Trimmomatic (24) in this step. Hisat2 was used to construct the index of the reference genome and aligned with clean reads at the end of the reference genome pair. Expression quantification of bam files was performed by StringTie and RNA-seq count data were obtained. The DESeq2 R package (25) was finally used as an expression analysis tool to analyze the differences between the two groups. The raw RNA-seq data have been deposited to GEO with an accession number GSE274164.

### Phosphoproteomics data analyses

For result processing, Protein Discoverer (Version 2.4, ThermoFisher Scientific) and the UniProt Human Protein Database (75,004 entries) were applied.

### Western blot analysis

The tissues were homogenized in 1% NP40 buffer^113^ containing phosphatase inhibitor cocktail (Yeasen Biotech) and protease inhibitor cocktail (APE×BIO) with metal beads. The lysates were then separated by SDS-PAGE and transferred to Immobilon 0.45 mm membranes (Cat# IPVH00010, Millipore). Western blotting was conducted using various specific primary and secondary antibodies. The membranes were incubated with diluted primary antibodies overnight at 4 °C and secondary antibodies for 1 h at room temperature. ECL Reagent (Cat# G2014-50ML, Servicebio) and ChemiScope machine (Cat# NO. 6000, Clinx Science Instruments Co., Ltd.) were used for the visualization of protein bands.

### Enzyme-linked immunosorbent assay (ELISA)

The serum samples were collected by centrifuging the whole blood cells of mice for 10 min at 4000 rpm and stored in a deep freezer. The levels of GDF15 protein in the serum were measured by an ELISA kit (Cat# MGD150, R&D Systems) following the manufacturer’s instructions. The adipose tissue samples were collected from mice fasting overnight and refeeding for 4 h. The amount of NTS was measured by an ELISA kit (Cat# CSB-EL016136MO, CUSABIO) following the manufacturer’s instructions.

### Human studies

All participants in both populations provided written informed consent. The cohort of children was described in the previous study,^114^ and the study has been approved by the ethics committee of Peking University Third Hospital (ethnic approval number 2021-283-06). In brief, 366 overweight or obese children were recruited from two elementary schools in Beijing, China. The basic clinical characteristics were collected by trained staff. The information about food intake was collected via a three-day 24-h dietary recall, and the nutrient composition was analyzed according to China Food Composition Tables by two independent investigators. The adult population consisted of 945 healthy individuals free of diabetes, gastrointestinal disease, cardiovascular diseases, cancer, or any use of medication in Shanghai, China, and this study has been approved by the Ethics Committees of Fudan University (FE20064) and Zhongshan Hospital (B2019-089R). The demographic traits of participants were collected and the habitual dietary information was collected using a validated food frequency questionnaire.^115^ For both populations, fasting blood samples were collected using EDTA anticoagulation tubes.

### Tissue lipid extraction

Lipid extraction was performed following a published protocol.^116^ For serum samples, they were centrifuged to remove cells at 4 °C, 14000× *g* for 10 min. 30 μL of serum, 120 μL of water, 1.5 mL of methanol and 5 mL of MTBE were added to a clean glass centrifuge tube, and 12 μL of lipid internal standards were added in each sample at the same time, including 2 μL of 0.82 mM PC (17:0/17:0), 2 μL of 0.77 mM PC (19:0/19:0), 2 μL of 1.40 mM PE (14:0/14:0), 2 μL of 4.91 mM LPC (17:0), 2 μL of 3.49 mM SM (d18:1/17:0) and 2 μL 4.53 mM of Cer (d18:1/17:0). The homogenate was vortexed for 1 min. The glass centrifuge tube containing the homogenate was rocked on a shaker for 1 h at room temperature. A total of 1.25 mL of water was then added to the glass centrifuge tube followed by another minute of vortexing. The homogenate was centrifuged at 4 °C, 1000× *g* for 10 min and two-phase layers could be observed in the glass centrifuge tube. A total of 4 mL of the top-phase supernatant was collected and dried under a stream of nitrogen. The extracted lipid samples were stored at –80 °C before LC-MS/MS analysis. For the adipose tissues, a 2 mg eWAT sample was added to 200 µL of water and 500 µL of methanol, and homogenized using the same approach as in the hydrophilic metabolite extraction above. The homogenate was supplemented with 500 µL more methanol and decanted into a clean glass centrifuge tube. 5 mL of MTBE was then added to the glass centrifuge tube and vortexed for 1 min. The glass centrifuge tube containing the homogenate was rocked on a shaker for 1 h at room temperature. 1.25 mL of water was then added to the glass centrifuge tube followed by 1 min of vortexing. The homogenate was centrifuged at 4 °C at 1000× *g* for 10 min and two-phase layers could be observed in the glass centrifuge tube. 4 mL of the top-phase supernatant were collected and dried under a stream of nitrogen. The extracted lipid sample was stored at –80 °C before MS analysis.

### Untargeted lipidomics

The untargeted lipidomics method was modified from a published method.^117^ Lipid samples were resuspended in 50 µL of isopropanol: acetonitrile:water (v/v/v, 30:65:5), and 10 µL was injected into Orbitrap Exploris 480 LC-MS/MS (ThermoFisher Scientific) coupled to HPLC system (Shimadzu, Kyoto, Japan). Lipids were eluted via C30 by using a 3 μm, 2.1 mm × 150 mm column (Waters) with a flow rate of 0.26 mL/min in buffer A (10 mM ammonium formate at a 60:40 ratio of acetonitrile:water) and buffer B (10 mM ammonium formate at a 90:10 ratio of isopropanol:acetonitrile). Gradients were held in 32% buffer B for 0.5 min and run from 32% buffer B to 45% buffer B at 0.5–4 min; from 45% buffer B to 52% buffer B at 4–5 min; from 52% buffer B to 58% buffer B at 5–8 min; from 58% buffer B to 66% buffer B at 8–11 min; from 66% buffer B to 70% buffer B at 11–14 min; from 70% buffer B to 75% buffer B at 14–18 min; from 75% buffer B to 97% buffer B at 18–21 min; 97% buffer B was held from 21–25 min; from 97% buffer B to 32% buffer B at 25–25.01 min; and 32% buffer B was held for 8 min. All the ions were acquired by non-targeted MRM transitions associated with their predicted retention time in a positive and negative mode switching fashion. ESI voltage was +5500 and −4500 V in positive or negative mode, respectively. All the lipidomics.RAW files were processed on LipidSearch 4.0 (ThermoFisher Scientific) for lipid identification. The lipidomic results were statistically analyzed with MetaboAnalyst 5.0^118^ and LINT-web.^119^

### Two-sample Mendelian randomization

Mendelian randomization studies use genetic variants that are associated with exposures to assess the causal associations of exposure with outcomes and aim to reduce bias from confounding and reverse causation.^120^ We applied a two-sample Mendelian randomization analysis to examine the potential causal association of circulating ceramides with food intake.

The instrumental variables of plasma levels of ceramides were obtained from the metabolic syndrome in men (METSIM) study.^121,122^ Fasting plasma levels of ceramides were measured using non-targeted metabolomics profiling in participants of the METSIM study. The genome-wide association study (GWAS) summary statistics of plasma ceramides levels were obtained from PheWeb Datasets (https://pheweb.org/metsim-metab/). GWAS summary statistics of daily consumption of food weight, energy, carbohydrate, fat and protein of the male participants from the UK Biobank (UKB) GWAS round 2 (http://www.nealelab.is/uk-biobank/) were used. There was no overlap of participants between the ceramides GWAS cohorts and the food intake GWAS cohorts. Further details regarding the GWAS summary data can be found in Supplementary information, Table S11.

In the initial step of analyses, exposure-related SNPs were selected for each ceramide (using a *P*-value threshold ≤ 5 × 10^−8^). We then conducted LD clumping for the instrumental variables with the TwoSampleMR (Version 0.5.6) package in R (R Version 4.2.1) to identify independent SNPs for each ceramide. We used a threshold of R^2^  <  0.001 to exclude dependent SNPs. Further information on the instrumental variables selected in relation to plasma ceramides levels can be found in Supplementary information, Table S12. We harmonized SNPs associated with the exposures (in this case, plasma ceramides levels) with SNPs associated with the outcomes (in this case, food weight, energy, carbohydrate, fat, and protein) using ‘harmonise_data’ function in TwoSampleMR package in R. All SNP data in this study are based on the GRCh37 reference genome coordinates. We excluded SNPs associated with outcomes in the METSIM study (*P* < 5 × 10^–8^) before Mendelian randomization analyses. Mendelian randomization analyses were performed using the ‘mr’ function in TwoSampleMR package in R. The inverse variance weighted method was used to meta-analyze their combined effects. Weighted median, simple mode, and weighted mode were used as sensitivity analyses.

To assess the possibility of reverse causality, reversal Mendelian randomization analyses were performed using the indicators related to food intake as exposures and the two ceramides as outcomes. LD-independent SNPs from corresponding GWAS that passed the same SNP filtering and proxy search steps mentioned previously were used as instrument variables. Due to the lack of significant SNPs, we were unable to perform the analysis for food intake.

### Targeted lipidomic analysis of ceramide

Samples were resuspended in 100 μL of isopropanol:acetonitrile:water (v:v:v, 65:30:5), and 10 μL was injected into a 6500 QTRAP triple-quadrupole MS (SCIEX) coupled to an HPLC system (Shimadzu). Ceramides were eluted via C30 by using a 3 μm, 2.1 mm × 150 mm column (Waters) with a flow rate of 0.26 mL/min using buffer A (10 mM ammonium formate at a 60:40 ratio of acetonitrile:water) and buffer B (10 mM ammonium formate at a 90:10 ratio of isopropanol:acetonitrile). Gradients were held in 32% buffer B for 0.5 min and run from 32% buffer B to 45% buffer B at 0.5–4 min; from45% buffer B to 52% buffer B at 4–5 min; from 52% buffer B to 58% buffer B at 5–8 min; from 58% buffer B to 66% buffer B at 8–11 min; from 66% buffer B to 70% buffer B at 11–14 min; from 70% buffer B to 75% buffer B at 14–18 min; from 75% buffer B to 97% buffer B at 18–21 min; 97% buffer B was held from 21–25 min; from 97% buffer B to 32% buffer B at 25–25.01 min; and 32% buffer B was held for 8 min.

Ceramides have similar precursor ions, such as d18:1 sphingosine (m/z 264 and 282), d16:1 sphingosine (m/z 236 and 254) and t18:0 sphingosine (m/z 282 and 300).^33^ All ions were acquired by 156 selected reaction monitoring transitions (Supplementary information, Table S15) just in a positive mode. Electrospray ionization voltage was +4900 V.

### Data representation and statistical analysis

The number of replicates were described in the figure legends. All the values in the figures are expressed as mean ± SEM. All the *P*-values were calculated via *t*-test with post-hoc correction unless specifically indicated.

## Supplementary information

Supplementary information, Figure Legends.

Supplementary information, Fig. S1

Supplementary information, Fig. S2

Supplementary information, Fig. S3

Supplementary information, Fig. S4

Supplementary information, Fig. S5

Supplementary information, Fig. S6

Supplementary information, Fig. S7

Supplementary information, Fig. S8

Supplementary information, Fig. S9

Supplementary information, Fig. S10

Supplementary information, Fig. S11

Supplementary information, Table S1

Supplementary information, Table S2

Supplementary information, Table S3

Supplementary information, Table S4

Supplementary information, Table S5

Supplementary information, Table S6

Supplementary information, Table S7

Supplementary information, Table S8

Supplementary information, Table S9

Supplementary information, Table S10

Supplementary information, Table S11

Supplementary information, Table S12

Supplementary information, Table S13

Supplementary information, Table S14

Supplementary information, Table S15
