# Supplementary material for: Neurotensin-neurotensin receptor 2 signaling in adipocytes suppresses food intake through regulating ceramide metabolism
Source: Cell Res. 2025 Jan 3;35(2):117–31. doi: 10.1038/s41422-024-01038-8 (PMC11770130; doi:10.1038/s41422-024-01038-8)
Supplement: Supplementary file 1 — Supplementary information, Figure Legends. [file 41422_2024_1038_MOESM1_ESM.docx]

**Figure legends**

**Supplementary information, Fig. S1: The elevation of thermogenesis in *Ntsr2* BKO mice.**

**a.** Establishment of brown/beige adipocyte specific *Ntsr2* KO mouse model; **b.** The KO efficiency and specificity of the *Ntsr2* gene; N=4-5; **c-d.** The body weight (**C**) and food intake (**D**) of mice fed by a chow diet. N=7; **e-n.** The body weight (**e**), body composition (**f**), GTT (**g**), ITT (**h**), O_2_ (**i**) or CO_2_ (**j**) production rate, the expression of thermogenic genes in BAT (**k**) or iWAT (**l**), fecal energy (**m**) and physical activity (**n**) of mice fed by HFD. N=7-8. *, p<0.05; **, p<0.01; ***, p<0.001. HY, hypothalamus; iWAT, inguinal white adipose tissue; eWAT, epidydimal white adipose tissue; BAT, brown adipose tissue.

**Supplementary information, Fig. S2: NTS-NTSR2 signaling inhibits the lipolysis of WAT.**

**a.** The KO efficiency in the floated adipocytes. N=5; **b.** The KO efficiency in the primary adipocytes. N=4-5; **c.** The body weight of mice fed by chow diet. N=9; **d.** The body weight of mice fed by HFD and kept in the thermoneutrality condition. N=4; **e.** The body composition of mice fed by chow diet. N=4; **f.** The expression of p-HSL and ATGL protein in the eWAT; **g.** The concentration of NEFA in the serum of control or *Ntsr2* AKO mice. N=8-10; **h-i.** The expression of p-HSL and ATGL protein (**h**) as well as NEFA secretion (**i**) from the primary adipocytes of the wild type mice upon NTS treatment. N=3; **j-k.** The expression of p-HSL and ATGL protein (**j**) as well as NEFA secretion (**k**) from the primary adipocytes of the *Ntsr2* AKO mice upon NTS treatment. N=3. *, p<0.05; **, p<0.01; ***, p<0.001.

**Supplementary information, Fig. S3: The impacts of NTS-NTSR2 signaling on metabolic homeostasis.**

**a-b.** The O2 consumption rate (**a**) and CO2 production rate (**b**) of the mice fed by HFD; N=4. **c.** The food intake of control and *Ntsr2* AKO mice fed by an HFD. N=8; **d.** The food intake of control and *Ntsr2* AKO mice fed by an HFD and kept in the thermoneutrality condition. N=4; **e.** The concentration of NTS peptide in different adipose tissues upon fasting and refeeding. N=4-5; **f.** The illustration of hydrogel containing NTS peptide; **g.** The illustration of NTS injection to the eWAT; **h.** The effects of NTS local release in the eWAT on the food intake of wild type mice. N=5-6; **i-j.** The effects of NTS local release on the body weight changes (**i**) and fat mass (**j**) of obese mice. N=6; **k-l.** The expression of *Nts* in the eWAT (**k**) and food intake (**l**) of control and *Nts* KO mice. N=8-9. *, p<0.05; **, p<0.01.

**Supplementary information, Fig. S4: The metabolism of ceramide controlled by the NTS-NTSR2 signaling in the WAT.**

**a.** The level of phosphorylated ERK (p-ERK) in the WAT of control and *Ntsr2* AKO mice; **b.** The level of phosphorylation for ER related proteins based on phosphor-proteomics data; N=3; **c.** The illustration of phosphorylation site for CerS2; **d.** The illustration of how to detect the phosphorylated CerS2 (p-CerS2) with the pull-down and western blot assay; **e.** The level of p-CerS2 with the treatment of CK2 inhibitor DMAT; **f-g.** The relative abundance of ceramide (**g**) or sphingosine (**g**) in the WAT, detected by the untargeted lipidomics. N=5-6; **h-i.** The concentration of ceramide in the iWAT (**h**) or BAT (**i**) of control and *Ntsr2* AKO mice, detected by targeted lipidomics. N=3; **j.** The concentration of ceramide in the primary adipocytes upon NTS treatment, detected by targeted lipidomics. N=3; **k-l.** The concentration of ceramide in the iWAT (**k**) or BAT (**l**) upon NTS treatment, detected by targeted lipidomics. N=4-5; **m.** The concentration of ceramide in the serum of control and *Ntsr2* AKO mice, detected by targeted lipidomics. N=5-6; **n.** The relative abundance of phosphorylated CERT (p-CERT) in the WAT. N=3. *, p<0.05; **, p<0.01; ***, p<0.001.

**Supplementary information, Fig. S5: CerS2 in the WAT regulated the metabolic homeostasis.**

**a.** The KO efficiency of *CerS2* detected by RT-qPCR. N=4; **b-c.** The concentration of ceramide C16-24 in the WAT (**b**) or serum (**c**) of control or *CerS2^+/-^* mice. N=4; **d.** The expression of *CerS5* and *CerS6* in the WAT. N=4; **e-f.** The concentration of ceramide C16-24 (**e**) as well as the expression of *CerS5* and *CerS6* (**f**) in the liver of control or *CerS2^+/-^* mice. N=4; **g.** The food intake of mice treated with ceramide C16. N=6; **h.** The intracellular concentration of C22 ceramide with 1-hour treatment of C22 ceramide. N=6; **i.** The expression of *CerS2* with the knocking-down of *CerS2* in the iWAT or eWAT locally. N=4-5; **j-k.** The concentration of ceramide C16-24 with the knocking-down of *CerS2* in the iWAT (**j**) or eWAT (**k**) locally. N=4-5. *, p<0.05; **, p<0.01; ***, p<0.001.

**Supplementary information, Fig. S6: NTS-NTSR2 signaling regulated UPR.**

**a.** The expression of UPR related genes in BAT. N=3-6; **b.** The expression of UPR related genes in the WAT of control or *Ntsr2* AKO mice upon pair-feeding. N=7-9; **c.** GSEA revealed the downregulation of UPR related genes in the WAT of control and *Ntsr2* AKO mice. N=3; **d-e.** The level of p-GCN2 (**c**) and p-PKR (**d**) in the WAT of control and *Ntsr2* AKO mice. N=3; **f.** The expression of two UPR related genes in the WAT of control and *Ntsr2* AKO mice. N=4; **g.** The expression of UPR related genes in the iWAT upon NTS treatment. N=4; **h.** The level of p-eIF2α in the primary adipocytes upon NTS treatment. N=3; **i.** The expression of UPR related genes in the eWAT of control or *Nts* KO mice. N=8; **j-k.** The expression of UPR related genes in the iWAT upon RhoA agonist (**j**) or antagonist (**k**) treatment. N=3-7. *, p<0.05; **, p<0.01; ***, p<0.001.

**Supplementary information, Fig. S7: Ceramide metabolism regulated UPR.**

**a-b.** The validation of overexpression (OE, **a**) and knocking-down (**b**) of *CerS2* in the primary adipocytes. N=4; **c.** The level of p-eIF2α in the primary adipocytes upon the overexpression of *CerS2*. N=3-4; **d.** The expression of UPR related genes in the primary adipocytes with *Ntsr2* AKO and knocking-down of *CerS2*. N=3; **e.** The expression of UPR related genes in the primary adipocytes with knock-down of *CerS2* upon NTS treatment. N=4-5; **f-g.** The expression of UPR related genes (**f**) and p-eIF2α (**g**) in the primary adipocytes upon ceramide C22 treatment. N=3-4; **h-i.** The expression of UPR related genes (**h**) and p-eIF2α (**i**) in the primary adipocytes upon ceramide C16 treatment. N=3-4; **j-k.** The expression of UPR related genes (**J**) and p-eIF2α (**K**) in the WAT of control or *CerS2^+/-^* mice. N=3; **l.** The expression of UPR related genes in the WAT upon ceramide C22 *in vivo*. N=3-4. *, p<0.05; ***, p<0.001.

**Supplementary information, Fig. S8: NTSR2 regulated food intake via GDF15.**

**a.** The abundance of GDF15 protein in the adipose tissue. N=3; **b.** The expression of *Gdf15* in the skeletal muscle of control or *Ntsr2* AKO mice. N=3-4; **c.** The serum concentration of GDF15 of control or *Ntsr2* AKO mice kept in the thermoneutrality condition. N=7-9; **d.** The serum concentration of GDF15 from the obese mice treated with NTS in the iWAT locally. N=4; **e.** The expression of *Gfral* in the AP/NTS region with or without *Gfral* knock-down. N=3; **f.** The food intake of mice with or without *Gfral* knock-down; N=12-14; **g.** The body weight changes compared to the day0 of the *Gfral* knocking-down in both control or *Ntsr2* AKO mice; N=3; **h-i.** The recombinant GDF15 treatment normalized the serum concentration of GDF15 (**h**) and food intake (**i**) in the control or *Ntsr2* AKO mice. N=4; **j-k.** The treatment of PERK agonist (PERKa) normalized the serum concentration of GDF15 (**j**) and food intake (**k**) in the control and *Ntsr2* AKO mice. N=7. *, p<0.05; **, p<0.01; ***, p<0.001.

**Supplementary information, Fig. S9: CerS2 is important for NTS-NTSR2 signaling.**

**a-b.** The relative expression of *Gdf15* upon RhoA antagonist (**a**) or agonist (**b**) treatment in the primary adipocytes. N=3-7; **c.** The relative expression of *Gdf15* upon NTS treatment in the primary adipocytes with knocking-down of *CerS2*; N=5; **d.** The relative expression of *Gdf15* upon the combinational treatment of NTS and RhoAa in the primary adipocytes. N=3-4; **e.** The production of GDF15 protein in the primary adipocytes with *Ntsr2* AKO and with or without *CerS2* overexpression; N=3-4; **f.** The expression of *Gdf15* gene in the WAT upon the treatment of ceramide C16 *in vivo*. N=3; **g.** The expression of *Gdf15* gene and GDF15 in the WAT upon the treatment of ceramide C22 *in vivo*. N=3-4; **h.** The abundance of GDF15 protein in the serum upon ceramide C22 treatment *in vivo*. N=3; **i.** The abundance of GDF15 protein in the serum upon knocking-down *CerS2* in the iWAT *in vivo*. N=3. *, p<0.05; **, p<0.01.

**Supplementary information, Fig. S10: The correlation between ceramide and physiological parameters in adults.**

**a-b.** The correlation between ceramide (d16:1_22:0) (**a**) or (d16:1_24:0) (**b**) and body weight, body mass index (BMI), fat mass, serum level of glucose, triglyceride (TG), total cholesterol (TC) and low-density lipoprotein cholesterol (LDL-c) in the adults.

**Supplementary information, Fig. S11. The correlation between ceramide and physiological parameters in teenagers.**

**a-b.** The correlation between ceramide (d18:1_22:0) (**a**) or (d18:1_24:0) (**b**) and body weight, body mass index (BMI), fat mass, serum level of glucose, triglyceride (TG), total cholesterol (TC) and low-density lipoprotein cholesterol (LDL-c) in the children.
