# Supplementary figures and images for: Neurotensin-neurotensin receptor 2 signaling in adipocytes suppresses food intake through regulating ceramide metabolism
Source: Cell Res. 2025 Jan 3;35(2):117–31. doi: 10.1038/s41422-024-01038-8 (PMC11770130; doi:10.1038/s41422-024-01038-8)

Fig. S1

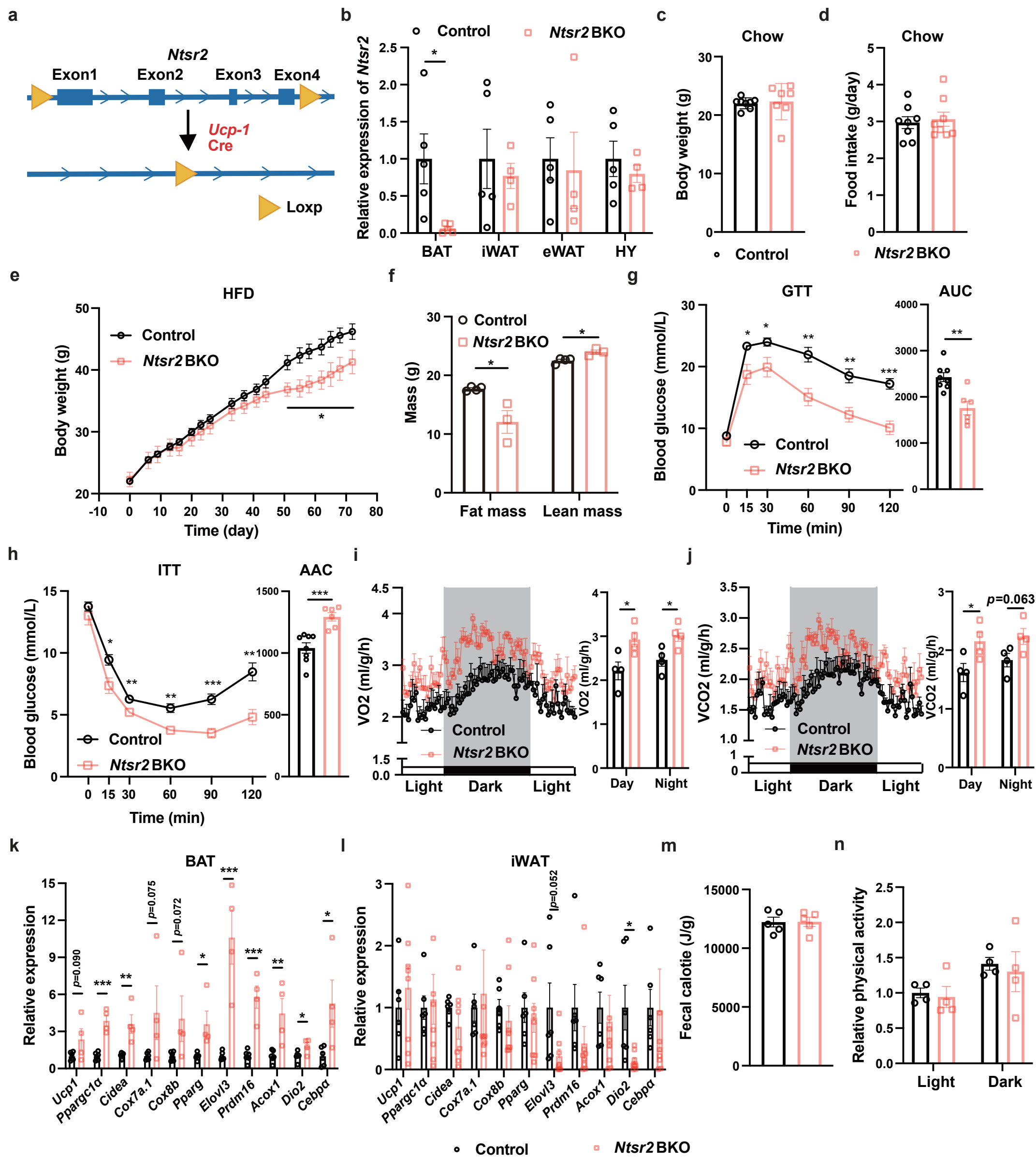

Supplement: Supplementary file 2 — Supplementary information, Fig. S1 [file 41422_2024_1038_MOESM2_ESM.pdf]

Fig. S2

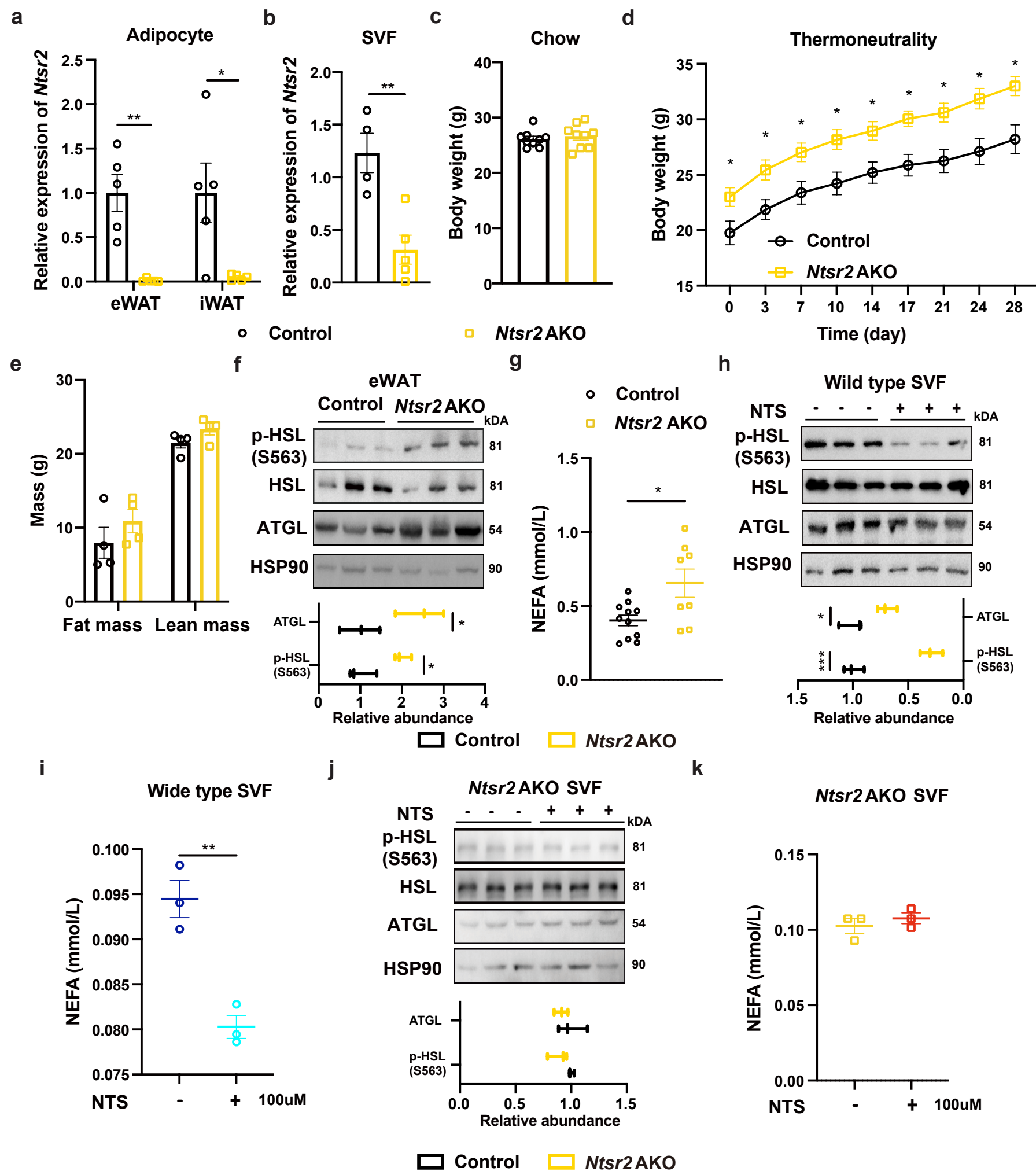

Supplement: Supplementary file 3 — Supplementary information, Fig. S2 [file 41422_2024_1038_MOESM3_ESM.pdf]

Fig. S3

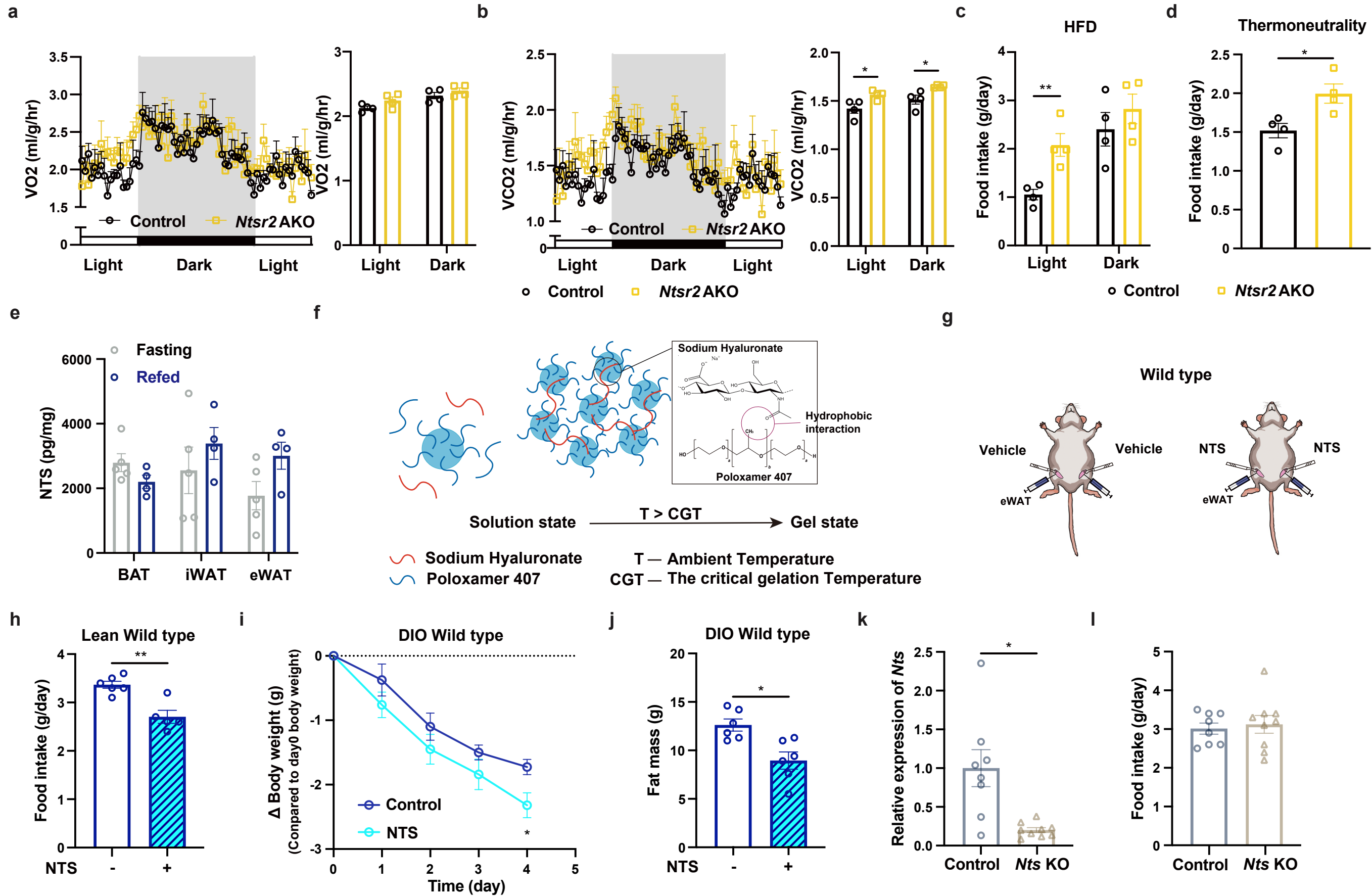

Supplement: Supplementary file 4 — Supplementary information, Fig. S3 [file 41422_2024_1038_MOESM4_ESM.pdf]

Fig. S4

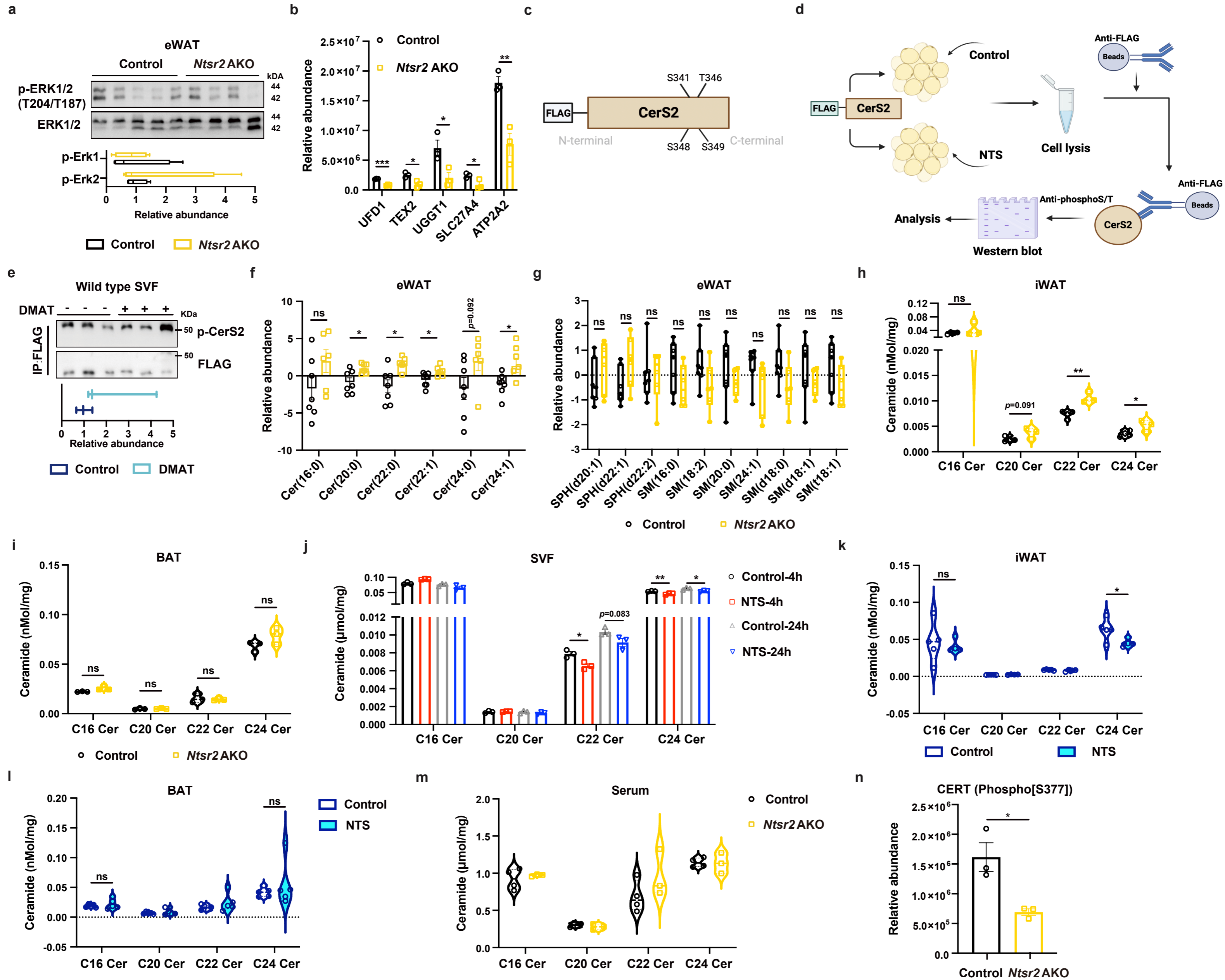

Supplement: Supplementary file 5 — Supplementary information, Fig. S4 [file 41422_2024_1038_MOESM5_ESM.pdf]

Fig. S5

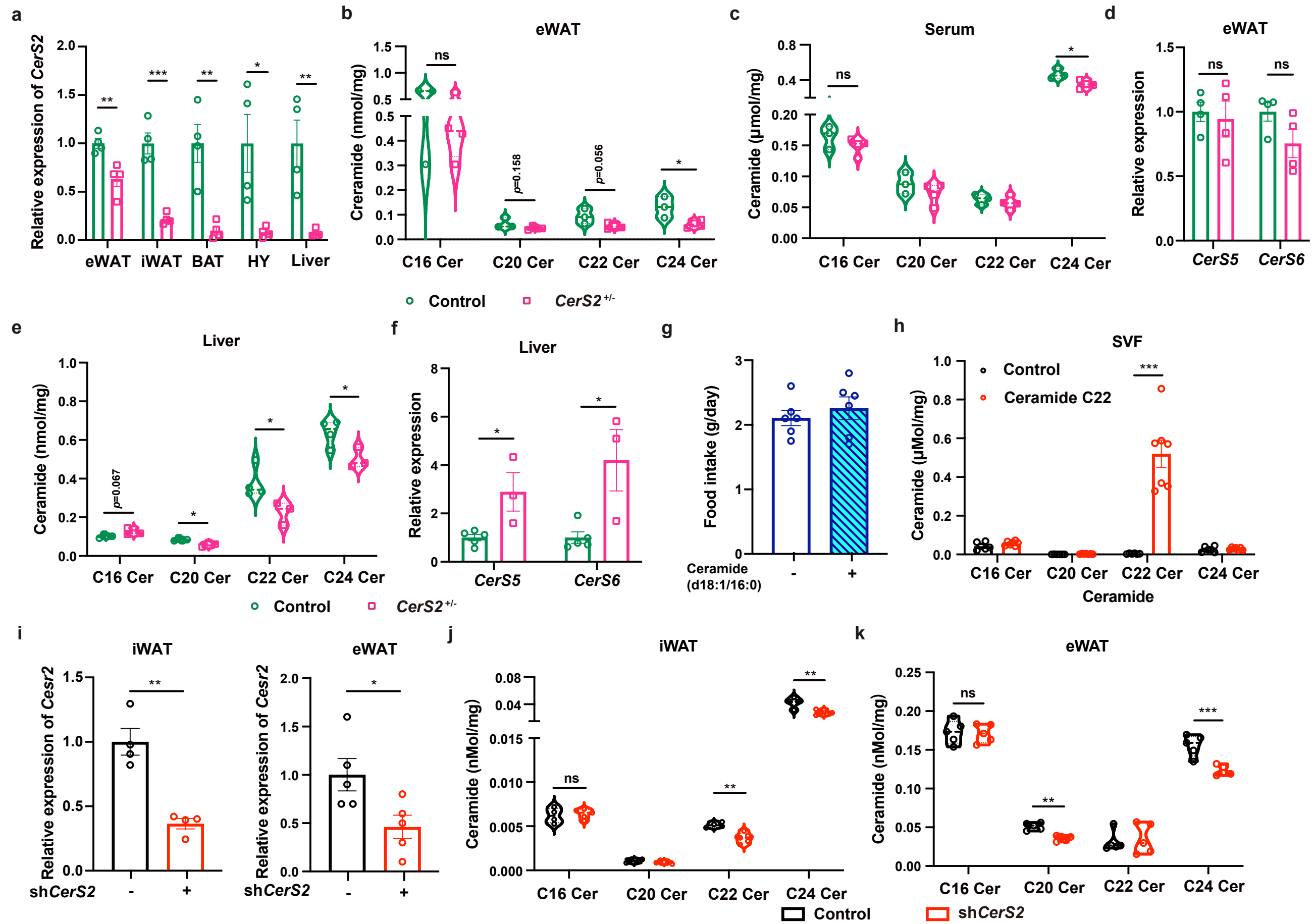

Supplement: Supplementary file 6 — Supplementary information, Fig. S5 [file 41422_2024_1038_MOESM6_ESM.pdf]

Fig. S6

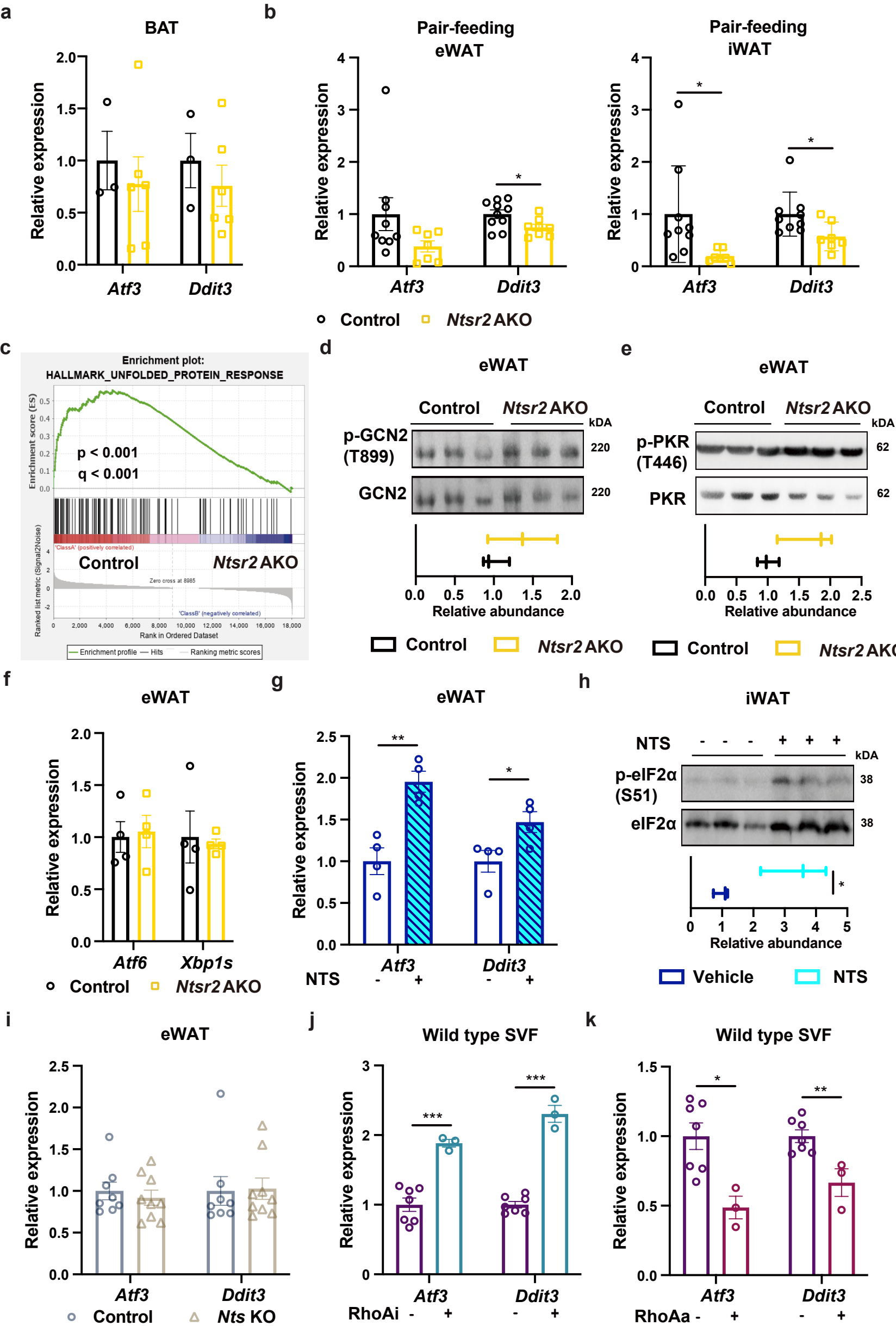

Supplement: Supplementary file 7 — Supplementary information, Fig. S6 [file 41422_2024_1038_MOESM7_ESM.pdf]

Fig. S7

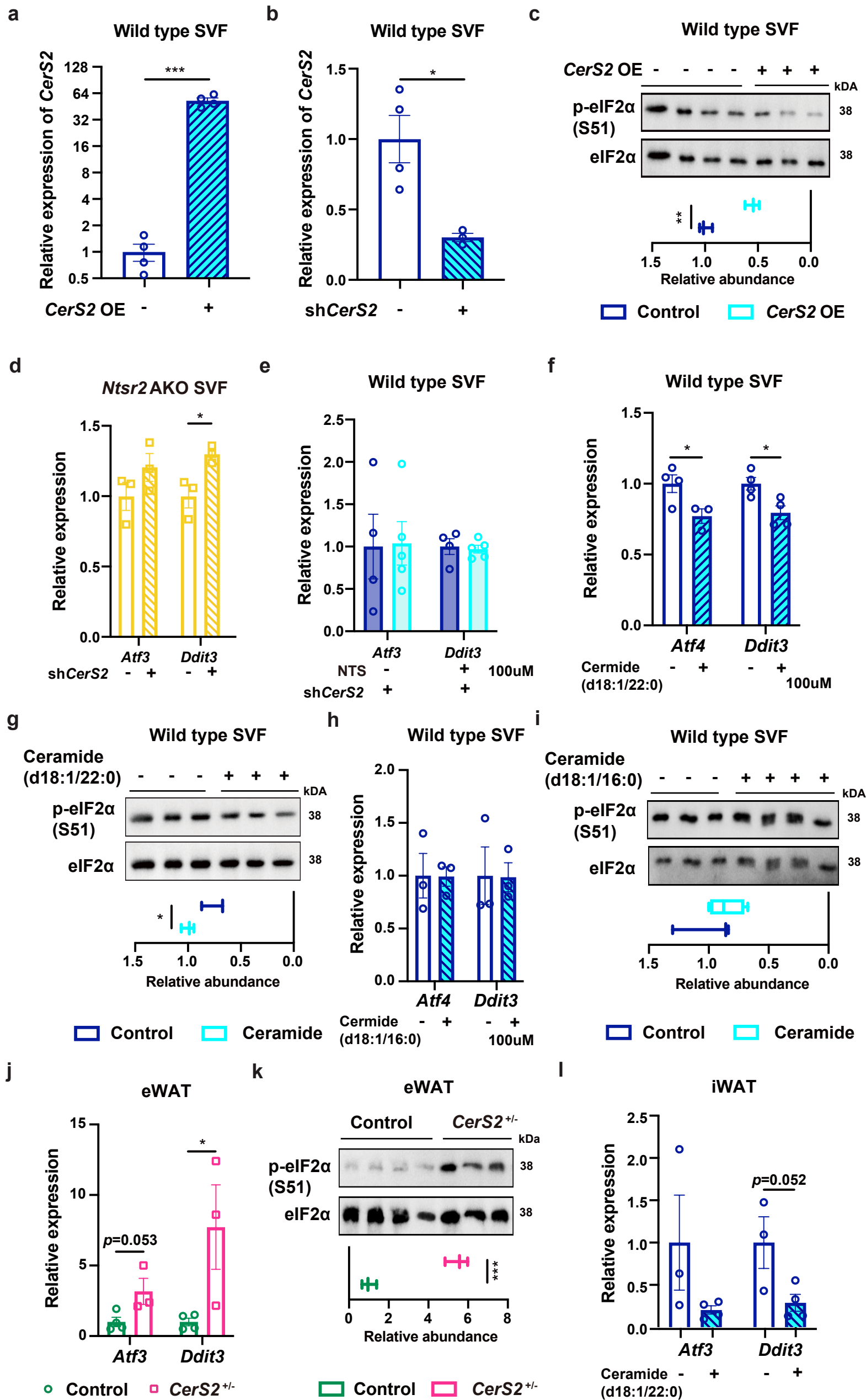

Supplement: Supplementary file 8 — Supplementary information, Fig. S7 [file 41422_2024_1038_MOESM8_ESM.pdf]

Fig. S8  
a

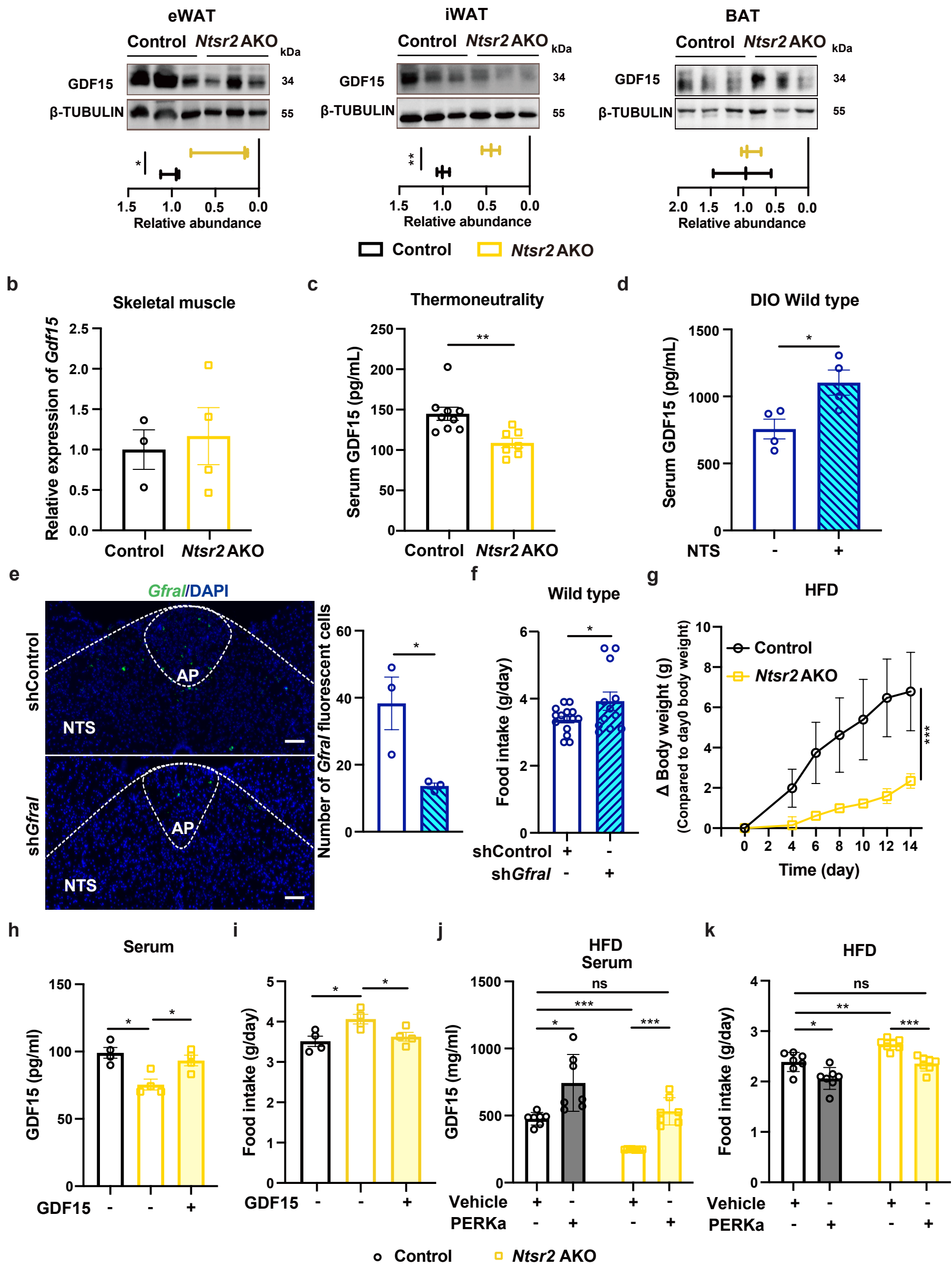

Supplement: Supplementary file 9 — Supplementary information, Fig. S8 [file 41422_2024_1038_MOESM9_ESM.pdf]

Fig. S9

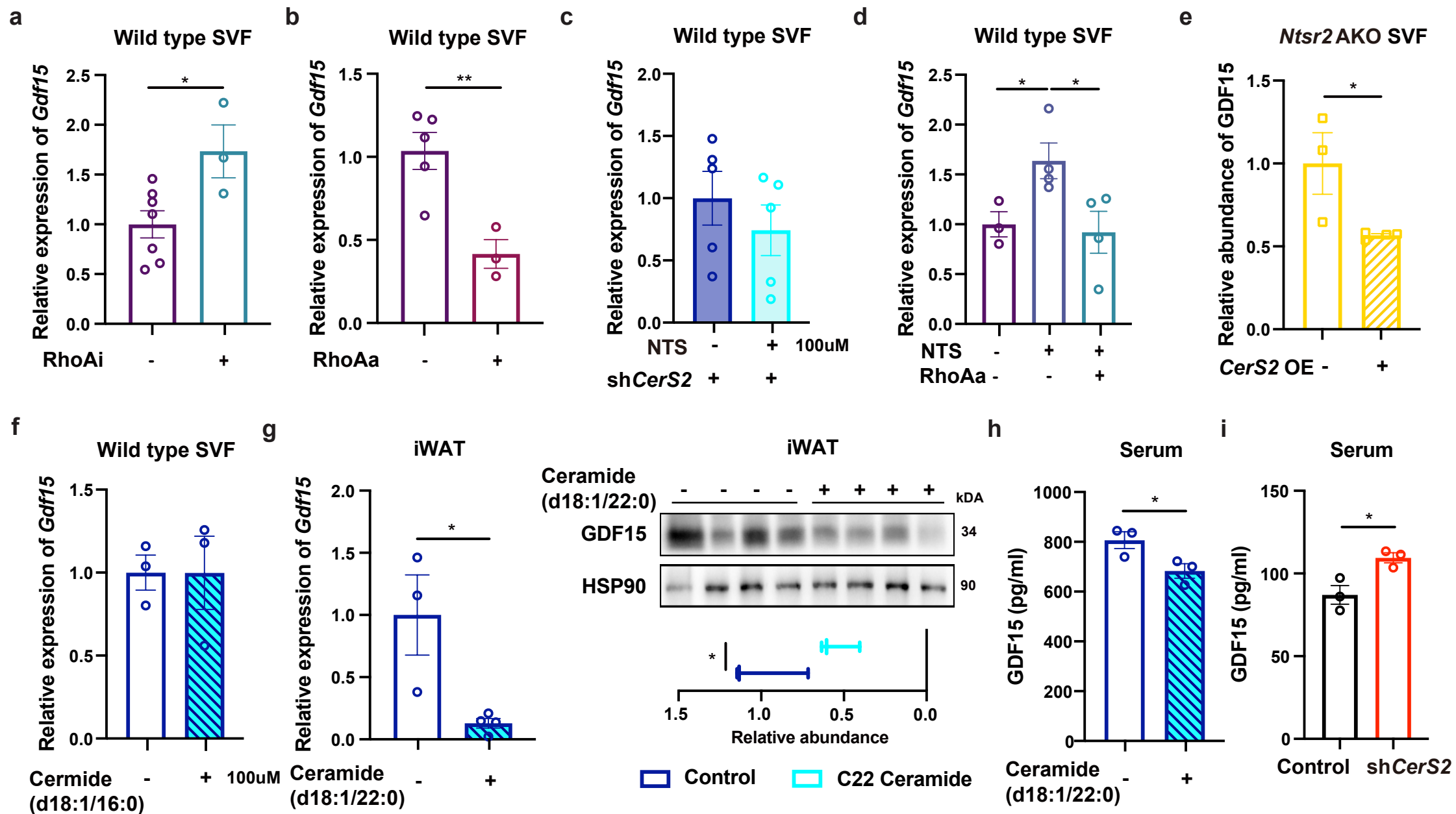

Supplement: Supplementary file 10 — Supplementary information, Fig. S9 [file 41422_2024_1038_MOESM10_ESM.pdf]

Fig. S10

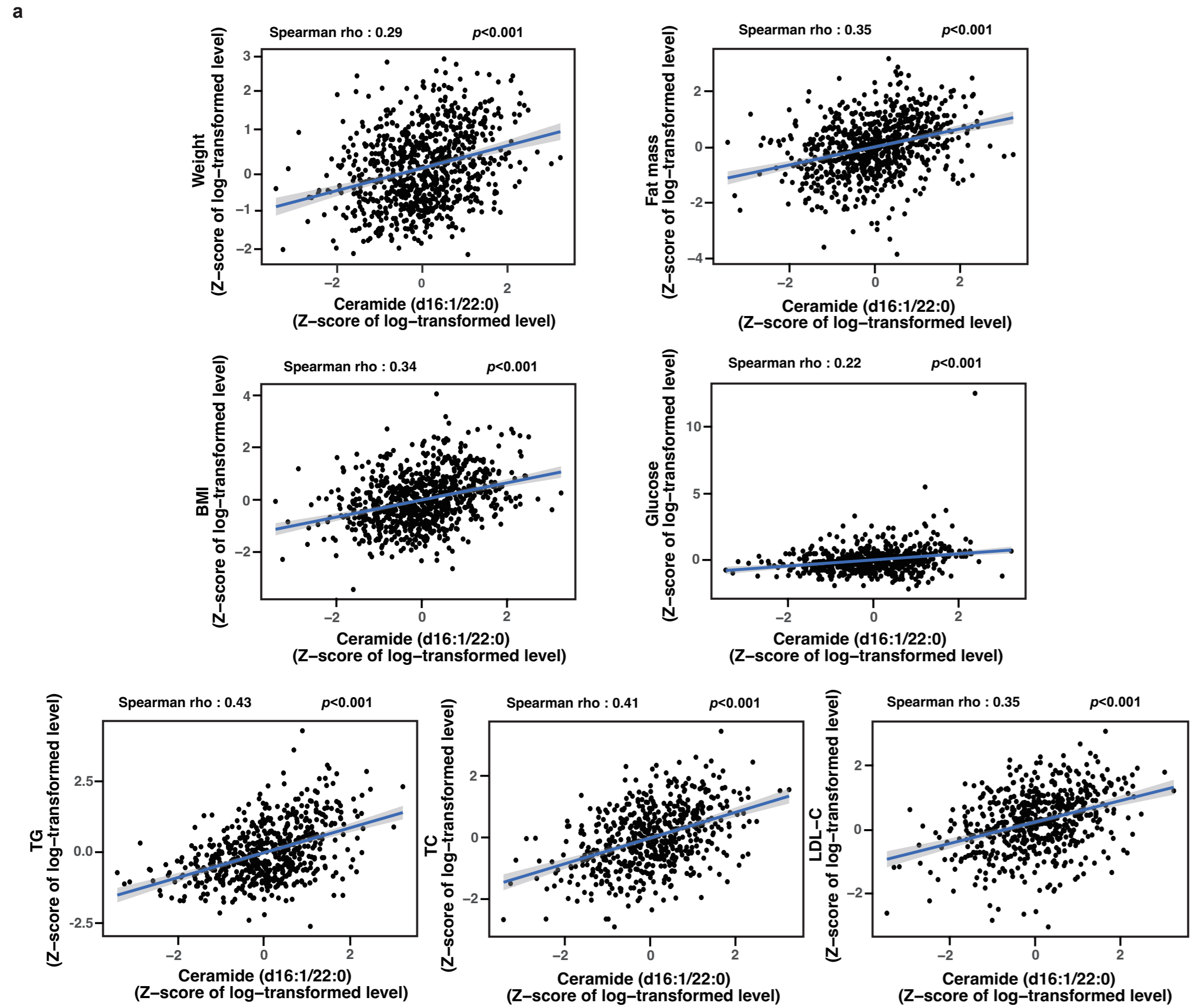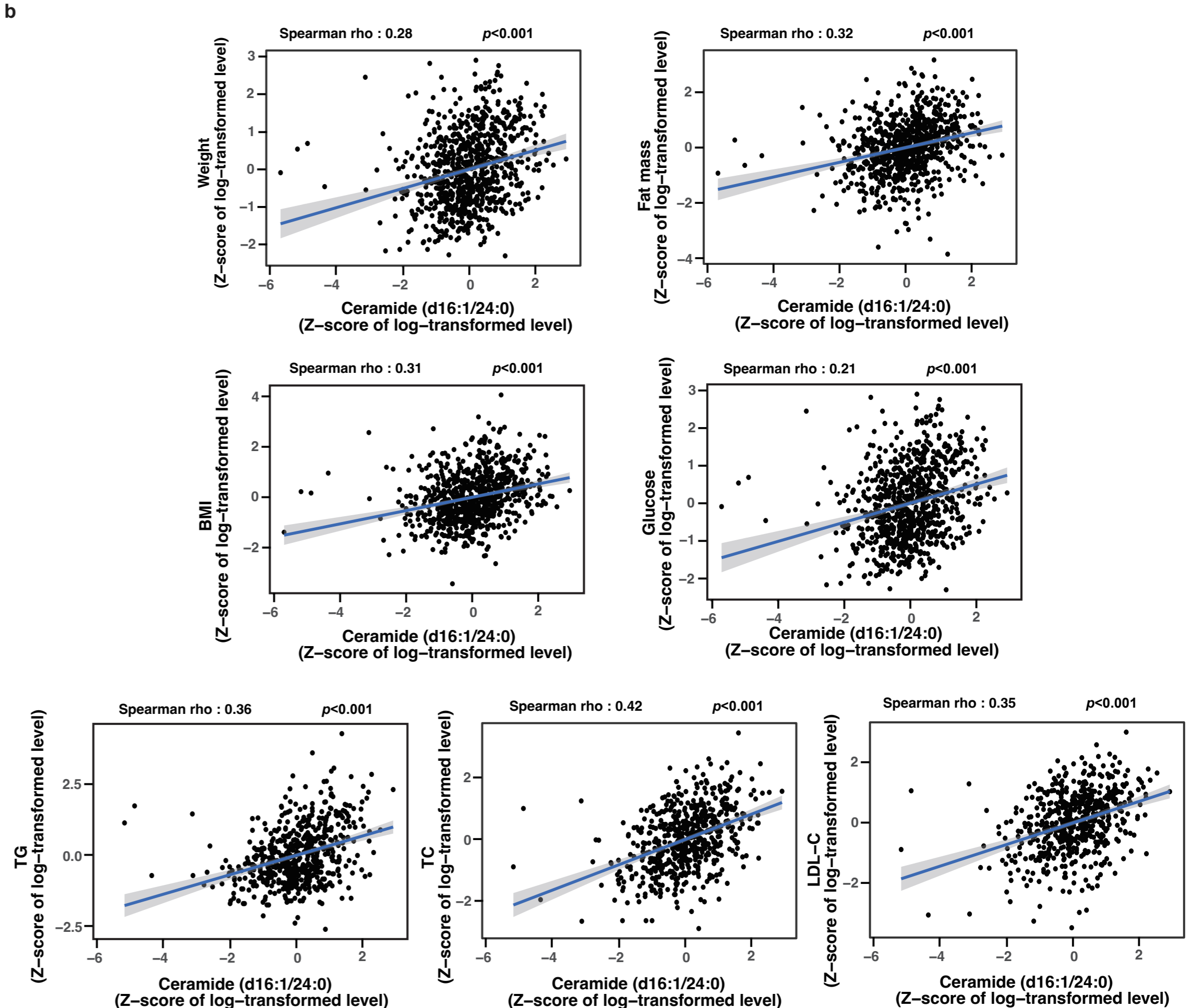

Supplement: Supplementary file 11 — Supplementary information, Fig. S10 [file 41422_2024_1038_MOESM11_ESM.pdf]

Fig. S11

a

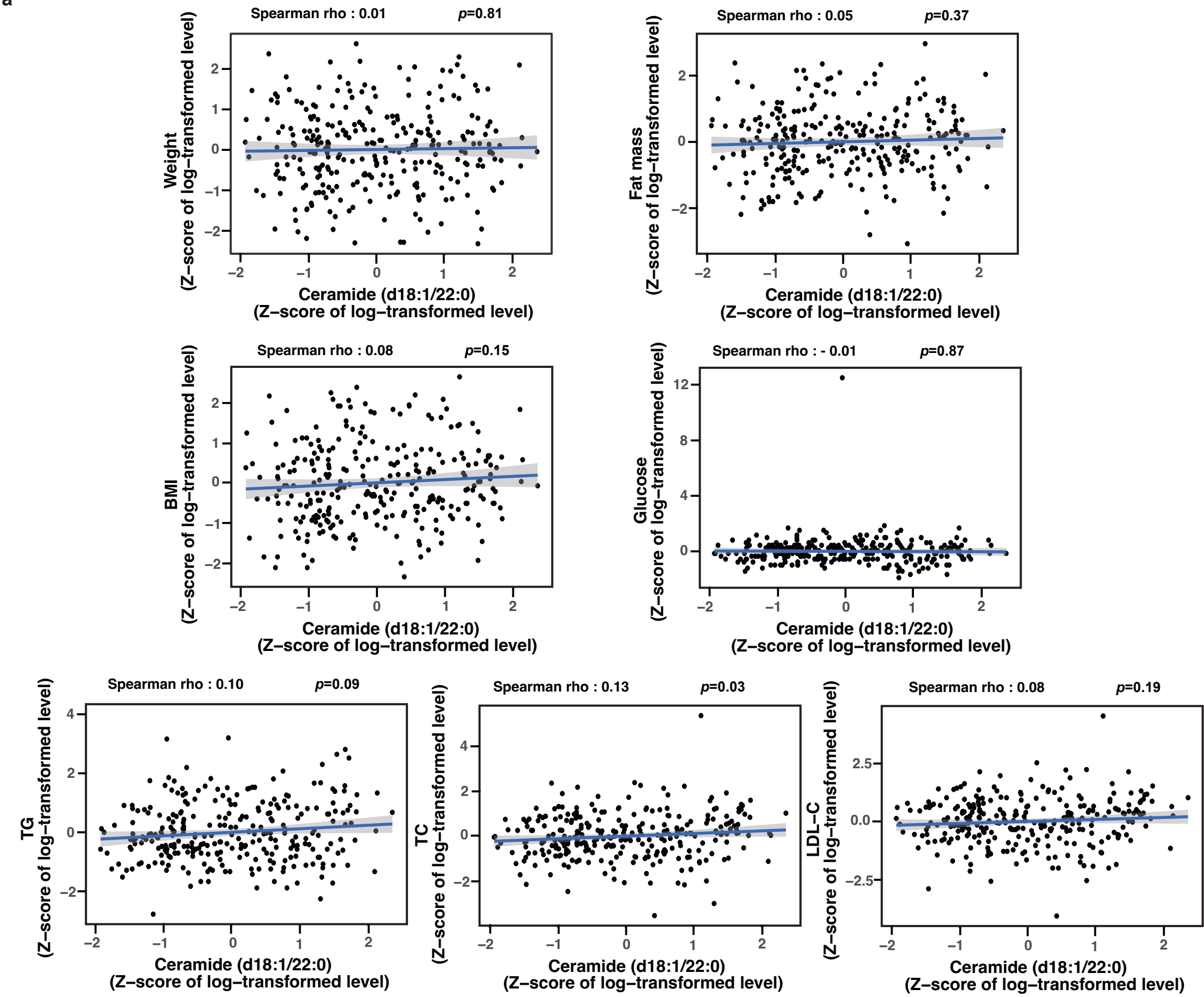

b

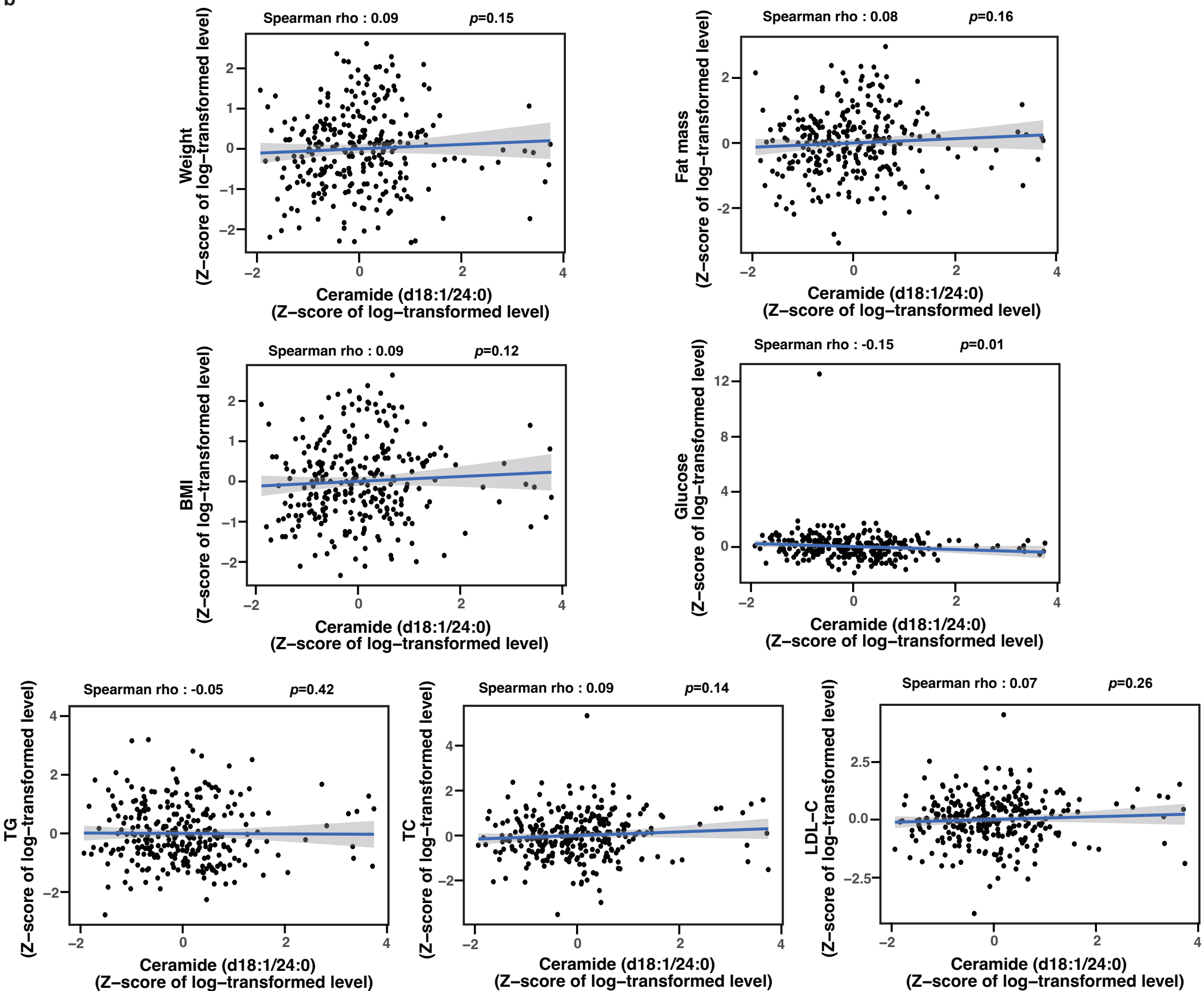

Supplement: Supplementary file 12 — Supplementary information, Fig. S11 [file 41422_2024_1038_MOESM12_ESM.pdf]
