# Supplementary material for: Neurotensin-neurotensin receptor 2 signaling in adipocytes suppresses food intake through regulating ceramide metabolism
Source: Cell Res. 2025 Jan 3;35(2):117–31. doi: 10.1038/s41422-024-01038-8 (PMC11770130; doi:10.1038/s41422-024-01038-8)
Supplement: Supplementary file 25 — Supplementary information, Table S13 [file 41422_2024_1038_MOESM25_ESM.docx]

**STable 13. KEY RESOURCES TABLE**

| REAGENT or RESOURCE | SOURCE | IDENTIFIER |
| --- | --- | --- |
| Antibodies |  |  |
| Rabbit monoclonal anti-eIF2α | Cell Signaling Technology | Cat# 5324S |
| Rabbit monoclonal anti-p-eIF2α (Ser51) | Cell Signaling Technology | Cat# 3398S |
| Rabbit monoclonal anti-PERK | Cell Signaling Technology | Cat# 3192S |
| Rabbit monoclonal anti-p-PERK (Thr980) | Cell Signaling Technology | Cat# 3179S |
| Rabbit polyclonal anti-GCN2 | Cell Signaling Technology | Cat# 3302S |
| Rabbit polyclonal anti-p-GCN2 (Thr899) | Thermo Fisher Scientific | Cat# PA5-105886 |
| Rabbit monoclonal anti-p-PKR (Thr446) | Abcam | Cat# Ab32036 |
| Rabbit monoclonal anti-PKR | Abcam | Cat# Ab184257 |
| Rabbit monoclonal anti-p-HSL (Ser563) | Cell Signaling Technology | Cat# 4139S |
| Rabbit monoclonal anti-HSL | Cell Signaling Technology | Cat# 18381S |
| Rabbit monoclonal anti-ATGL | Cell Signaling Technology | Cat# 2138S |
| Mouse GDF-15 Antibody | R&D Systems | Cat# AF6385 |
| Rabbit monoclonal anti-beta-Tubulin | ABclonal | Cat# AC008 |
| Mouse monoclonal anti-HSP90 | Abcam | Cat# Ab203085 |
| Phospho-(Ser) 14-3-3 Binding Motif | Cell Signaling Technology | Cat# 9606 |
| Anti-Flag-Tag monoclonal Antibody | GNI GROUP, Inc. | Cat# GNI 4310-FG |
| Goat Anti-Rabbit IgG(H+L)/HRP | Bioss Antibodies | Cat# bs-40295G-HRP |
| Goat Anti-Mouse IgG(H+L)/HRP | Bioss Antibodies | Cat# bs-40296G-HRP |
| Bacterial and virus strains |  |  |
| AAV9-GFRAL shRNA | Vigene Biosciences | N/A |
| AAV9-pscAAV-U6-GFP | Vigene Biosciences | N/A |
| AAV9-*Cers2* shRNA | OBiO Technology | N/A |
| Chemicals, peptides, and recombinant proteins |  |  |
| Mouse/Rat GDF-15 Quantikine ELISA Kit | R&D Systems | Cat# MGD150 |
| NTS ELISA Kit | CUSABIO | Cat# CSB-EL016136MO |
| Isobutylmethylxanthine | Sigma | Cat# I5879 |
| Collagenase D | Roche | Cat# 11088858001 |
| Insulin from bovine pancreas | Sigma | Cat# I6634 |
| Dexamethasone | Sigma | Cat# D4902 |
| Dimethyl Sulfoxide | Sigma | Cat# D2660 |
| Rosiglitazone | Sigma | Cat# R2408 |
| C22-Ceramide (d18:1/22:0) | MedChemExpress | Cat# HY-154831 |
| C12-Ceramide | MedChemExpress | Cat# HY-100353 |
| CCT020312 | MedChemExpress | Cat# HY-119240 |
| Forskolin | MedChemExpress | Cat# HY-15371 |
| Trizol | Thermo Fisher Scientific | Cat# 15596018 |
| PrimeScript™ RT reagent Kit | Takara | Cat# RR037A |
| TB Green® Premix Ex Taq™ | Takara | Cat# RR420B |
| PEG300 | MedChemExpress | Cat# HY-Y0873 |
| Tween80 | MedChemExpress | Cat# HY-Y1891 |
| α-D-Glucose | Sigma | Cat# 158968 |
| Neurotensin | MedChemExpress | Cat# HY-P0234 |
| Bovine serum albumin | BBI Life Sciences | Cat# A600332-0100 |
| NON-Fat Powdered Milk | BBI Life Sciences | Cat# A600669-0250 |
| Phosphatase Inhibitor Cocktail | Yeasen Biotech | Cat# 20109ES20 |
| Protease Inhibitor Cocktail | APE×BIO | Cat# K1007 |
| Carprofen | Selleckchem | Cat# S4136 |
| Sodium Chloride for Injection 10ml | Hanna Pharmaceutical | Cat# NC9054335 |
| DMEM high glucose | Gibco | Cat# C11965500BT |
| RIPA buffer | Servicebio | Cat# G2002 |
| 10x PBS | Servicebio | Cat# G4207 |
| Paraformaldehyde | Servicebio | Cat# G1101 |
| Anti-Fade Mounting Medium | BBI Life Sciences | Cat# E675011-0010 |
| Trypsin/EDTA 0.25% | Gibco | Cat# 25200-056 |
| Nonesterified Free fatty acids assay kit | Nanjing Jiancheng Bioengineering Institute | Cat# A042-2-1 |
| BCA Protein Assay Kit | Beyotime | Cat# No. P0012 |
| Mouse Direct PCR Kit | Bimake | Cat# B40015 |
| Oil Red solution | Servicebio | Cat# G1016 |
| Hematoxylin solution | Servicebio | Cat# G1004 |
| Differentiation solution  (With 60% ethanol as solvent) | Servicebio | Cat# G1039 |
| Scott Tap Bluing | Servicebio | Cat# G1040 |
| Glycerin gelatin sealed tablets | Servicebio | Cat# G1042 |
| OCT embedding agent | Servicebio | Cat# G6059 |
| Sirius Red solution set | Servicebio | Cat# G1018 |
| Sucrose | Sinopharm Group Chemical Reagent | Cat# 57-50-1 |
| Corning™ Regular Fetal Bovine Serum | Gibco | Cat# MT35010CV |
| Water HPLC | Fisher Chemical | Cat# W6-4 |
| Acetonitrile | Fisher Chemical | Cat# A998-4 |
| 2-Propanol HPLC (99.9%) | Fisher Chemical | Cat# A464-4 |
| Methanol HPLC (99.9%) | Fisher Chemical | Cat# A452-4 |
| Formic acid | Fisher Chemical | Cat# A117-50 |
| Acetic Acid HPLC (99.9%) | Fisher Chemical | Cat# A35-500 |
| Ammonium Hydroxide | Fisher Chemical | Cat# A470-250 |
| MTBE HPLC (99.9%) | Sigma Aldrich | Cat# 650560-1 |
| SDS-PAGE gel preparation kit | Servicebio | Cat# G2003 |
| ACK lysing buffer | Gibco | Cat# A10492-01 |
| Ceramide (d18:1/22:0) | Sigma | Cat# 860501 |
| GDF15 recombinant protein | Abcam | Cat# ab202199 |
| Neurotensin peptides | This paper | Pyr-LYENKPRRPYIL |
| Poloxamer-407 | BASF | Cat# 9003-11-6 |
| Sodium Hyaluronate | Shandong Focusfreda Biotech Co., Ltd. | Cat# HA-E2.3 |
| DMAT | MedChemExpress | Cat# HY-15535 |
| C16-Ceramide | MedChemExpress | Cat# HY-100354 |
| CCG-1423 | APE×BIO | Cat# B4897 |
| U 46619 | APE×BIO | Cat# B6890 |
| Midostaurin | APE×BIO | Cat# B3709 |
| Prostratin | APE×BIO | Cat# C4359 |
| Salirasib | APE×BIO | Cat# A3787 |
| 7-Fluorotryptamine hydrochloride | MedChemExpress | Cat# HY-117295A |
| JW67 | MedChemExpress | Cat# HY-108442 |
| HLY78 | MedChemExpress | Cat# HY-122816 |
| Fragment (6-22) amide TFA | MedChemExpress | Cat# HY-P1290A |
| 6-Bnz-cAMP sodium salt | APE×BIO | Cat# B7791 |
| ZCL278 | APE×BIO | Cat# A8300 |
| LY294002 | APE×BIO | Cat# A8250 |
| 740 Y-P | APE×BIO | Cat# B5246 |
| Anti-FLAG M2 Magnetic Beads | Sigma | Cat# M8823 |
| Experimental models: Organisms/strains |  |  |
| Mouse: C57BL/6J | GemPharmatech | Cat# N000013 |
| Mouse: *Adipoq-cre* | Jax Lab | Cat# 028020 |
| Mouse: *Ucp1-cre* | Jax Lab | Cat# 024670 |
| Mouse: *Prox1-cre/ERT* | Jax Lab | Cat# 022075 |
| Mouse: *Ntsr2^flox/flox^* | This paper | N/A |
| Mouse: *Nts^flox/flox^* | This paper | N/A |
| Mouse: *Cers2*^+/-^ | This paper | N/A |
| Oligonucleotides |  |  |
| Genotyping primers: *Ntsr2* flox :5’- GCAAAGCTGCTTCTCTTTACTGAG-3’; 5’- AGATAGATGGACCTCAAAGGCAG-3’; *Adipoq*-cre: 5’- ACGGACAGAAGCATTTTCCA-3’; 5’- GGATGTGCCATGTGAGTCTG-3’；*Ucp1*-cre：5’- GTCCTGGAACGTCATCATGTTTG -3’; 5’- GCTTCCTTCACGACATTCAACAG -3’；*Cers2^+/-^*: 5’- TTCATCCACAAGAGCAGTGACCAG -3’; 5’- AAGTCCTCACCTTCAAAGCAAGC -3’; | This paper | N/A |
| Supplementary Table 14 | This paper | N/A |
| Other |  |  |
| HFD diet (60%) | ResearchDiet | Cat# D12492 |
| Blood Glucose meter | OneTouch UltraEasy meter | N/A |
| Blood Glucose Strips | OneTouch | N/A |
| NTS ELISA Kit |  |  |
| Cell strainer | Corning | Cat# 352340 |
| Immobilon 0.45mm membranes | Millipore | Cat# IPVH00010 |
| Software and algorithms |  |  |
| Protein Discoverer | Thermo Fisher Scientific | Version 2.4 |
| Prism | GraphPad Version 8.0 | N/A |
| ImageJ | ImageJ Software | Version 1.8.0 |
| DESeq2 | R studio | Version 4.0 |
